# Inflammation, epigenetics, and metabolism converge to cell senescence and ageing: the regulation and intervention

**DOI:** 10.1038/s41392-021-00646-9

**Published:** 2021-06-28

**Authors:** Xudong Zhu, Zhiyang Chen, Weiyan Shen, Gang Huang, John M. Sedivy, Hu Wang, Zhenyu Ju

**Affiliations:** 1 Key Laboratory of Ageing and Cancer Biology of Zhejiang Province, Institute of Ageing Research, Hangzhou Normal University School of Medicine, Hangzhou, China; 2 Key Laboratory of Regenerative Medicine of Ministry of Education, Guangzhou Regenerative Medicine and Health Guangdong Laboratory, Institute of Ageing and Regenerative Medicine, Jinan University, Guangzhou, China; 3 Division of Pathology and Experimental Hematology and Cancer Biology, Cincinnati Children’s Hospital Medical Center, Cincinnati, OH USA; 4 Department of Molecular Biology, Cell Biology and Biochemistry, Brown University, Providence, RI USA

**Keywords:** Medical research, Cell biology

## Abstract

Remarkable progress in ageing research has been achieved over the past decades. General perceptions and experimental evidence pinpoint that the decline of physical function often initiates by cell senescence and organ ageing. Epigenetic dynamics and immunometabolic reprogramming link to the alterations of cellular response to intrinsic and extrinsic stimuli, representing current hotspots as they not only (re-)shape the individual cell identity, but also involve in cell fate decision. This review focuses on the present findings and emerging concepts in epigenetic, inflammatory, and metabolic regulations and the consequences of the ageing process. Potential therapeutic interventions targeting cell senescence and regulatory mechanisms, using state-of-the-art techniques are also discussed.

## Introduction

Individual cells face three major cell fate choices: to survive, to senesce, or to commit suicide. The balance between these processes ensures that cell turnover in an organism remains essentially in functional equilibrium (homeostasis). While cell survival and death have been intensively investigated for long, cell senescence is relatively less understood due to the complexity of the ageing process and heterogeneity of ageing phenotypes.^1^ Past investigations of cell senescence have focused on its role in tumor suppression, cell cycle arrest, tissue repair, and DNA replicative stress/damage response. Many mediators are linking to intrinsic (e.g., organelle homeostasis, chronic inflammation, and epigenetic alterations) and extrinsic factors (e.g., UV exposure, drug toxicity, and lifestyle) that deteriorate cell physiology.^2^ We now know many pathways involve in the regulation of cell senescence, such as AMP-activated protein kinase (AMPK) energy-sensing,^3^ histone/protein (de-)acetylation,^4,5^ cyclic GMP–AMP synthase (cGAS)–the cyclic GMP–AMP receptor stimulator of interferon genes (STING) signaling pathways,^6^ which impact the rate and extent of cell senescence. Of note, existing studies define that senescence as a relatively inert, non-proliferating, irreversible cell state.^1^ Although the removal of senescent cells serves as an attractive option to mitigate age-related functional decline and extend health span,^7^ researchers are trying to reprogram senescent cells back to functionally healthy cells, especially for cardiomyocytes and neurons that are hardly proliferative. Can cell senescence be reversed? Are there different forms or stages of senescent cells that exert distinct physiological effects? Does senescence have any yet new functions to the cells or tissues? To our knowledge, there are still many open questions that await answers.

**Fig. 1:**
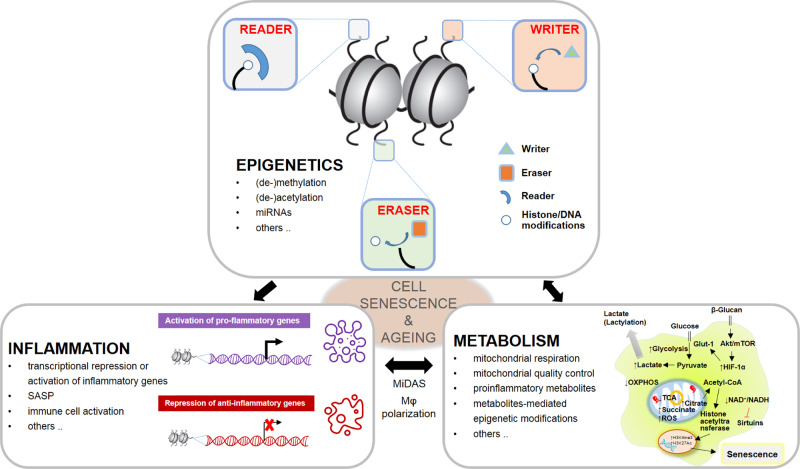
Dysregulated inflammation, alteration of epigenetic modifications, and metabolic imbalance converge to cell senescence and ageing. Cross talks among epigenetic modifiers (writers, readers, and erasers), inflammatory gene expression and immune cell response, and metabolic alterations contribute to senescent phenotypes and organ degeneration. MiDAS mitochondrial dysfunction-associated senescence, SASP senescence-associated secretory phenotype, OXPHOS oxidative phosphorylation, ROS reactive oxygen species, TCA tricarboxylic acid

As the biological techniques advance, many new targets and signaling pathways participating in cell senescence regulation have been identified, although some are still controversial. Nonetheless, the common signals and mechanisms converge upon dysregulated inflammation, alteration of epigenetic modifications, and metabolic imbalance.^8–10^ Here in this review, we highlight those known and yet unexploited key molecular and signaling pathways, particularly the inflammatory, epigenetic, and metabolic aspects that link cell senescence and organismal ageing (Fig. 1). Therapeutic targets and novel techniques established in recent cell senescence studies are also discussed.

## Definition of cell senescence

**Fig. 2:**
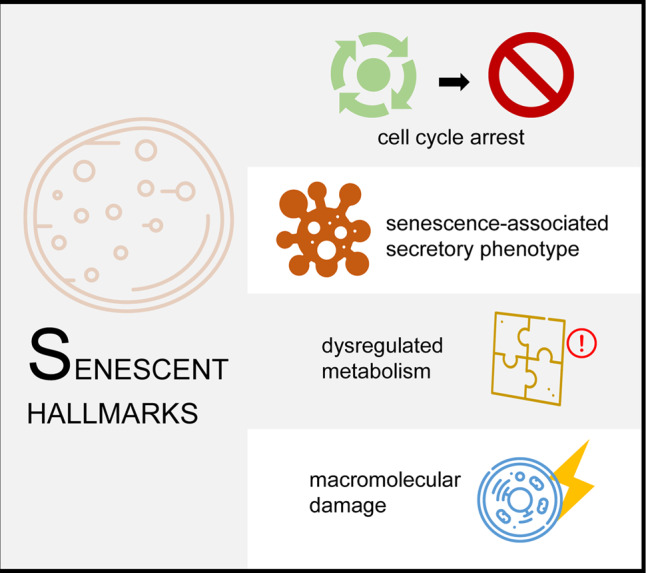
The hallmarks of cell senescence

Although research on cell senescence lasts for decades, until recently, the field reached a consensus on the definition of cell senescence, that is, a type of cell state that can be stimulated by multiple stress signals throughout the life cycle and is characterized by cell cycle arrest, senescence-associated secretory phenotype (SASP), and dysregulated metabolism and macromolecular damage^1^ (Fig. 2). Consequently, many types of cell senescence have been proposed, including replicative senescence, programmed developmental senescence, and stress-induced senescence.^11^ Such diverse senescent pathways represent active and passive modes to establish a delicate balance between different cell populations—as abstract as it sounds. It is worth noting that ageing and cell senescence are different but closely related. Organismal ageing emphasizes the degeneration of tissues or organs caused by accumulated damages upon a period.^12^ In contrast, cell senescence can occur whenever under a specific stress condition, and it may also play a positive role in wound healing and tumor inhibition.^13^ The determinant that matters favorable or lousy cell senescence largely relies on the duration: long-term type of cell senescence is prone to inflammation and disease, while short-term type sometimes seems beneficial because the immune system can quickly scavenge the senescent cells.^14^

## The hallmarks of cell senescence

As described above, senescent cells are categorized by differences in stress stimuli, thereby exhibiting various phenotypes and hallmarks. At present, the most commonly used senescent marker is senescence-associated beta-galactosidase (SA-β-gal), closely related to the lysosomal stress response, but not necessarily dependent on senescence.^15^ Activation of p53, p21CIP1, p16INK4a, ataxia telangiectasia mutated (ATM)/ATM and RAD3-related (ATR), and retinoblastoma (RB) can be used as auxiliary markers apart from the occurrence of morphological changes, permanent cell cycle arrest, cell secretion, and metabolic and chromatin remodeling.^16^ As none of those mentioned above markers can solely determine the specific type of cell senescence, various methods and hallmarks are required to clarify the exact category of a senescent cell.^17^ A summary of known hallmarks of cell senescence is shown in Table 1.

**Table 1:** Selected modulators of cell senescence

Senescent modulators	Circumstances	References
SA-β-gal-positive staining	Development, ageing, all stages	^15^
p21CIP1	Cell cycle arrest, ageing, all stages	^568^
p16INK4a	Cell cycle arrest, ageing, all stages	^569^
p53	Cell cycle arrest, ageing, DNA damage	^570^
Rb	Cell cycle arrest, ageing, DNA damage	^571^
Lamin B1	Decrease in ageing, genome instability	^572^
TAFs/TIFs	DNA damage, telomere attrition	^573,574^
Phospho-γH2AX	DNA damage	^575^
SASP components	Ageing, DAMPs, all stages	^18^
Mitochondrial dysfunction	Decrease respiration and ATP production	^576^
Protein aggregates	UPR, ageing, loss of proteostasis	^577^
Oxidative stress	Increase ROS/RNS	^576^
Autophagy malfunction	Ageing, protein toxicity	^578^
Macromolecular condensates	Various stress, ageing	^19^
HP1	Various stress, SAHF	^579^
DNA methylation	Various stress, SAHF	^580^
Histone methylation	Various stress, SAHF	^581^
Morphology	Ageing	^582^
Multi-omics alteration	Various stress, ageing	^583^

### Permanent cell cycle arrest

Typically, senescent cells exhibit permanent cell cycle arrest that differs from quiescent or differentiated cells. The current definition of cell senescence emphasizes a state of cell cycle withdrawal upon external stimuli.^1^ In contrast, quiescent cells can reenter the cell cycle, and differentiated cells can be dedifferentiated under certain circumstances. Besides, senescence typically accompanies augmented p21CIP1 or p16INK4a expression, while quiescence is p27KIP1 dependent, and differentiation can involve multiple signaling pathways (e.g., Wnt/Notch/Hedgehog/p16^INK4a^).

### Senescence-associated secretory phenotype

First discovered by Jean-Philippe Coppe and colleagues in 2008, SASP refers to a state when senescent cells release certain substances that mediate a series of (patho-)physiological effects, including pro-inflammatory cytokines, chemokines, growth hormones, angiogenic factors, and matrix metalloproteinases.^18^ The different biological activities induced by the components of SASP suggest that it may interact with local and surrounding cells, and constitutes a mechanism to regulate microenvironment, which could be either beneficial or deleterious, depending on the secretion factors, site (cell types), duration (acute or chronic), and secretion-induced stimuli.

### Dysregulated metabolism

Metabolic disturbance during cell senescence manifests the loss of molecular and protein homeostasis. Several processes, such as DNA damage response (DDR) induced by telomere attrition, decreased tricarboxylic acid cycle activity, and ATP production by mitochondrial dysfunction, declined degradation of the proteasome and autophagolysosome, the changes in SASP and epigenetic alteration, all lead to the remodeling of metabolic signals and metabolites in the cells.

### Macromolecular damage

Biomacromolecular damage is another typical phenotype of cell senescence. Stimuli-like ionizing radiation, chemotherapeutics, and other drug damage, oxidative stress, and senescence-induced telomere dysfunction can mediate such damage, subsequently leading to the aggregation of aberrant macromolecular substances.^19^ Of note, the emerging concept of liquid–liquid phase separation in cell senescence has recently made the research of biomacromolecule condensate in the ascendant.

## Effects of cell senescence on organ function and diseases

Accumulating studies have proven the relationship between senescent cells and organismal ageing. Meanwhile, the concept of eliminating senescent cells to counteract ageing-related conditions has emerged and succeeded in rodent models. Baker and colleagues have found a large number of p16INK4a-positive senescent cells in various tissues that cause a range of ageing symptoms, including sarcopenia, cataracts, and lipodystrophy.^7^ Accordingly, targeted clearance of p16INK4a senescent cells alleviates the adverse symptoms and successfully extend the health span in many diseased models.^7^

The field began to look for traces of senescent cells in common ageing diseases in humans, and successfully established a causal relationship between pathogenesis of ageing-related diseases and cell senescence. Take atherosclerosis as an example, we have known that plaques composed of fat and protein gradually accumulate on the inner arterial wall, which is prone to cause coronary atherosclerotic disease, stroke, or other ischemic severe diseases. Next, senescence-associated macrophages were recruited to the arterial wall, where the plaque initially formed. As time elapsed, other senescent cell types appeared near these sites. Compared with other control cells, these senescent cells expressed abundant secretory factors and metabolites that promoted the pathogenesis of atherosclerosis, concurrent with significant alterations in epigenetic imprints.^20^ Using a variety of approaches to remove these senescent cells attenuated the lesions, and thus alleviating the progress of atherosclerosis.^20^ Consequently, focusing on the epigenetic and immunometabolic regulation of cell senescence may shed light on managing ageing-related diseases and therapeutic interventions.^21^

In this review, we highlight the recent advances in the understanding of the inflammatory, epigenetic, and metabolic basis of cell senescence, a comprehensive overview of relevant molecules and signaling pathways associated with cell senescence and organismal ageing are discussed. Finally, novel techniques and strategies intervening in the ageing process are briefly summarized.

## Ageing and inflammation

Infection-related diseases are responsible for many deaths globally, and aged people are more vulnerable to severe and life-threatening infections.^22,23^ Chronic inflammation represents an essential phenomenon in both murine and human ageing.^24^ Recent studies from different aspects investigations conclude that inflammation is a commonly shared mark of ageing tissues. Gene comparisons of young and old tissues from mice, rats, and humans revealed that age-related gene expression changes most remarkably involve a strong induction of inflammation and immune response genes.^25^ The combination of high levels of inflammatory signals, increased comorbid conditions, and reduced function of the immune system increases the vulnerability of aged individuals to infection.^26^

Despite its crucial role in defending infections during life, inflammation may turn into a hazardous factor to health for the aged individuals, when it gets into chronic and persistent, such chronic increases in low-grade inflammation during ageing, also known as “inflammageing”, is a hallmark of ageing.^16,27^ Unlike acute inflammation, inflammageing is characterized by maintaining a low-grade, sustained background of inflammation even in the absence of acute infection and clinically diagnostic disease.^27,28^ Increasing evidence has been shown that inflammageing is a risk factor that leads to reduced tissue repair and generative capacity, which is associated with many ageing-related diseases.^29–31^

Consequently, extending the understanding of the underlying mechanism of inflammageing and ageing-associated diseases helps realize healthy ageing among the growing senile population. In this section, we summarize the current findings illustrating the cause and effect of inflammageing, and how inflammation gives rise to the evolution of ageing-related diseases. Novel approaches to attenuate pathophysiological conditions are also discussed, aiming at combating adverse inflammageing.

### Source of chronic inflammation in ageing

High levels of pro-inflammatory cytokines shape the ageing-associated pro-inflammatory status, although the source of ageing-related chronic low-grade inflammation remains incompletely understood.^24,32–34^ Nevertheless, a variety of source that contributes to the pro-inflammatory microenvironment has been identified.^35^

#### Senescent cells

**Fig. 3:**
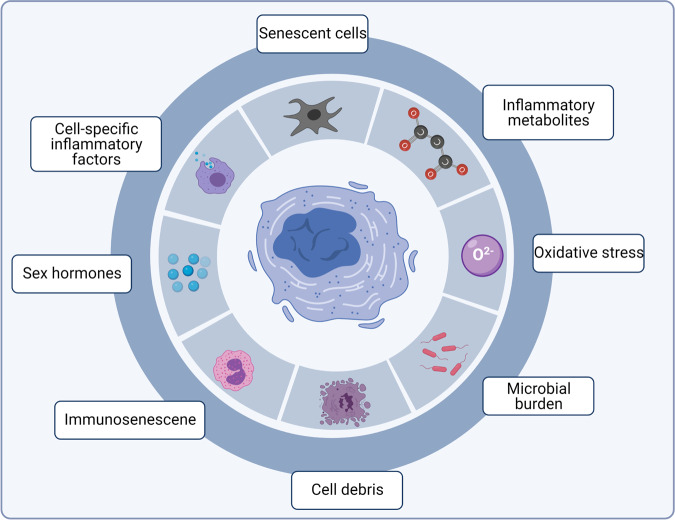
Inflammatory sources lead to senescence-associated secretory phenotype (SASP). Several pro-inflammatory sources have been identified to trigger the chronic inflammation during cellular senescence and organismal ageing, featured by the activation of a group of SASP-related genes, and subsequently the release of SASP components

Cells are driven into a senescent, nondividing state by many factors, such as telomere shortening, DNA damage, oxidative stress, genotoxic stress, and altered chromatin structure.^36–39^ The immune system efficiently clears away the senescent cells to maintain systemic homeostasis. However, the removal capacity declines with age (partially due to the immunosenescence), resulting in the increased SASP. Therefore, it becomes more explicit that SASP builds a relationship connecting cellular senescent with various biological processes, where SASP represents a potential pharmaceutical target to manipulate the development of ageing and ageing-related diseases (Fig. 3). In agreement, mounting evidence suggests that therapeutic removal of senescent cells could delay or even prevent various age-related diseases, such as atherosclerosis and osteoarthritis.^7,20,40,41^

#### Cell debris

Cell debris, including damage-associated molecular patterns (DAMPs), damaged organelles, and macromolecules, are recognized and removed by the immune system. With age, cell debris accumulates due to impaired clearance and overproduction, inducing augmented inflammation and impairs tissue regeneration.^42,43^ The ageing-associated mitochondrial compromise can lead to a release of DAMPs, namely the secretory phenotype by mitochondrial dysfunction-associated senescence (MiDAS), which causes particular attention in recent years.^44–47^ Congruently, ageing-associated mitochondrial stress can also lead to aberrant activation of inflammasomes and result in a functional decline in aged hematopoietic stem cells (HSCs), further stressing the immune system.^48^ Intriguingly, a study found that the level of circulating mitochondrial DNA (mtDNA) is significantly increased in elderly individuals and contributes to the increased systemic inflammation, although the exact source for circulating mtDNA remains undefined.^49^

#### Immunosenescence

The innate immune system gradually overtakes the adaptive immune system during ageing.^50^ In general, the function of the immune system declines with age, collectively termed immunosenescence, featured by the reduced output of natural killer cells and thymic T cells, decreased phagocytic capacity of macrophages, as well as the impaired activation of neutrophils and maldevelopment of B cells in the aged.^50,51^ Immunosenescence has been considered a dominating problem in the aged population and is associated with inappropriate immune responses, resulting in the declined removal capability of senescent cells and DAMPs.^52^ Reciprocally, inflammageing leads to chronic, continuous generation of inflammatory factors that exhausts the adaptive immune responses, culminating with immunosenescence.^10^ Of note, due to the suppression of the adaptive immune response, the innate immune response could be reinforced as a compensatory means to protect the organism from infections. Thus, the immunosenescence and inflammageing could operate in parallel and form a vicious feedback loop.

#### Gut dysbiosis

The gut mucosa barrier plays a vital role in defending against bacterial invasion. However, the integrity of the gut is impaired with age. The permeability of the epithelial cells is damaged, allowing the bacteria and other toxins to enter the blood, called “leaky gut”.^53–55^ The gut microbiota of older people also exhibits a biased diversity.^53–55^ For instance, reduced anti-inflammatory bacteria like *Bifidobacterium* spp., while increased pro-inflammatory bacteria like *Streptococcus* spp. was found in the aged gut.^56^ These changes lead to an increase in the susceptibility to infections in aged people.^57^ Another study also shows that gut microbiota links to longevity, in which healthier seniors showed microbiome signatures that are similar to young people.^58^ In line with this, the long-living (>90 years old) individuals favor increased gut microbiota diversity than that in young controls, with several beneficial bacteria identified in the gut.^59^

#### Obesity

Obesity is featured by excessive fat accumulation that secret many inflammatory adipokines.^60,61^ During ageing, immune cells infiltrate into the fat tissues that can be activated upon various stimuli. Bernier and colleagues recently demonstrated that anti-inflammatory Disulfiram, an FDA-approved drug treating chronic alcohol addiction, reversed established diet-induced obesity and metabolic dysfunctions in middle-aged mice.^62^ Thus, bodyweight control or calorie restriction (CR) that eliminates pro-inflammatory fat deposition would reduce inflammation during ageing.

#### Sex hormones

Existing evidence shows that sex steroids regulate the immune system by expressing their specific receptors in different immune cells.^63^ With age, the levels of sex hormones, such as estrogen and progesterone in females and testosterone in males are downregulated.^64–66^ Interestingly, after menopause, the number of lymphoid cells decreases, accompanied by a strong induction of pro-inflammatory cytokines.^67–69^ In contrast, postmenopausal females receiving hormone replacement therapies (HRT) showed increased B cells and reduced concentration of pro-inflammatory cytokines compared with that without HRT.^69,70^ Despite that testosterone replacement therapy has not been reported with aged male individuals, one study using old nonhuman primates clearly showed that supplementation of androgens in aged male rhesus macaques partially reverted the reduced number of naive T cells via enhancing thymic output, implicating a possible connection between age-related hormone dysregulation and immune dysfunction.^71^

#### Other sources

Apart from the sources discussed above, several lifestyle-related factors affect the secretory phenotypes of inflammageing.^72^ First, long-term smoking has been associated with the increased susceptibility of respiratory diseases, and especially lung cancer in the elderly, with a significantly elevated production of pro-inflammatory cytokines, such as interleukin (IL)-1, IL-6, TNF-alpha, and acute phase proteins.^73,74^ Second, a sedentary lifestyle among the aged individuals also accelerates fat accumulation and myeloid-biased hematopoiesis, siding with the pro-inflammatory microenvironment. In agreement with that, a recent study found that regular exercise activity results in the reduced inflammatory cell production, limiting the secretion of the inflammatory cytokines via modulating hematopoietic and progenitor cell proliferation in both murine and humans.^75^ Similarly, sleep problem perplexes aged individuals that aids mental stresses with elevated circulating inflammatory cytokines.

### Mechanisms involve in inflammageing

Although the mechanism of inflammageing has not been thoroughly studied, many factors include oxidative stress, pro-inflammatory cytokines, DNA damage, dysfunction of cellular organelles, defects in autophagy, and stem cell ageing are involved in regulating inflammageing at both transcriptional and posttranscriptional levels.^76^

#### Cytokines induction

Pathogen-associated molecular pattern receptors, such as the toll-like receptors (TLRs) expressed on immune cells, are the principal receptors that sense pathological stimuli and lead to cytokine induction. TLRs are the first to be affected by invading pathogens and mediate a series of physiological reactions, such as inflammation, cell survival, proliferation, and apoptosis.^77^ During ageing, the activation of TLRs downstream signaling pathways is altered.^78,79^ Among the transcription factors that regulate chronic inflammation across multiple diseases and tissues, NF-kB (nuclear factor kappa-light-chain enhancer of activated B cells) and STAT (signal transducer and activator of transcription) are the two well studied.^80^ NF-kB positively regulates many genes that encode pro-inflammatory cytokines, therefore acting as a master regulator of SASP.^81–83^ Moreover, NF-kB drives several ageing phenotypes, particularly in the skin, spine, brain, and blood system.^84–87^ Notably, mTOR controls the translation of IL-1a and thus regulates SASP, indicative of its role in the regulation of SASP.^88,89^ mTOR also has been manifested to control the translation of MK-2 kinase, which phosphorylates the specific RNA-binding protein ZFP36L1, preventing the degradation of the transcripts of many SASP factors.^89^ These findings lead to the assumption that mTOR accumulation helps accelerate the synthesis of SASP factors. Moreover, the surroundings of senescent cells and their communications also contribute to the SASP, for instance, the NOTCH/JAG1 signaling controls the interaction between senescent cells with their microenvironment.^90,91^

#### Oxidative stress-induced inflammageing

Based on the close relationships between oxidative stress, inflammation, and ageing, De La Fuente and Miquel proposed an oxidation-inflammatory theory of ageing (oxi-inflammageing).^92^ That is, oxidative stress leads to inflammageing and influences the homeostasis of the body. The redox state and the function of immune cells affect the velocity of ageing and life span.^92^ Therefore, antioxidants treatment may improve immune function. In line with this, resveratrol and metformin supplementation could extend life span via reducing oxidative stress.^93,94^

#### DNA damage response

DNA damage induces several signaling transductions that result in damage repair, cell cycle arrest, apoptosis, and cell death.^95,96^ Apart from the responses mentioned above, DNA damage also triggers cellular senescent and ultimately induces SASP.^97^ p38 is the primary regulator of DDR, and its activation could induce NF-kB signaling, causing the SASP-related gene expression.^98,99^ Studies showed that p38 inhibition prevents the secretion of various inflammatory factors involved in SASP.^100,101^ DNA damage also leads to the imbalance of systemic metabolism via inducing tissue inflammation.^102^ It should be noted that DNA damage accumulated during ageing, potentially contributing to the increase in chronic inflammation with age.

#### Cytosolic double-strand DNA-induced inflammageing

Viral DNA in cytoplasm triggers the cGAS–STING pathway, leading to the interferon (IFN) production and subsequently activates inflammatory response.^103–105^ Cytoplasmic DNA released from stressed mitochondria or damaged nuclei can also lead to innate immune signaling response via inflammasome or cGAS signaling.^106,107^ The cGAS–STING signaling also connects genomic instability and DNA damage to inflammation.^108^ Accumulated evidence suggests that endogenous retroelements, such as short interspersed nuclear elements (SINEs; including Alu) and long interspersed nuclear elements (LINEs; including LINE1), play an essential role in initiating inflammation.^109–111^ De Cecco and colleagues demonstrated that LINE1 was transcriptionally activated in senescent cells, thereby leading to type-I interferon (IFN-I) induction and promoting SASP.^112^

#### Micro-RNAs

The intracellular signaling cascades that regulate inflammageing are subject to numerous layers of regulation, including the regulation by micro-RNAs.^113,114^ For example, miRNAs participate in modulating TLR, retinoic acid-inducible gene I (RIG-I), and NF-kB signaling pathways.^115–118^ miRNA can directly bind on TLR signaling or activate the RNA-sensing TLRs. In turn, the expression of miRNA can also be regulated by TLRs, RIG-I, and NF-kB activation, revealing a feedback loop controlling the immune response.^117,119–121^

#### Stem cell ageing

Stem cells underlie tissue homeostasis, while ageing causes a functional decline in the stem cells, compromising tissue regeneration and contributing to age-related degenerative diseases.^122^ Chronic inflammation is one of the main factors that induce stem cell ageing.^123^ During ageing, aberrant activation of the NLRP3 inflammasome restraints the function of HSCs.^48^ Likewise, inflammageing is the main culprit of skeletal stem and progenitor cell dysfunction.^124^ The chronic inflammatory process accompanied by ageing leads to dysfunctional differentiation of stem cells, loss of self-renewal capacity, and results in stem cell ageing.^123^ Conversely, stem cell ageing is also responsible for systematic inflammageing. An increase in NF-kB activity has been reported in aged HSCs, leading to enhanced sensitivity in aged HSC to inflammatory stimuli, which result in the higher production of IL-6 and a myeloid-biased differentiation.^87,125^ Mesenchymal stromal cells (MSCs) ageing leads to adipocytes accumulation in old bones and dysregulates hematopoiesis.^126,127^ A detailed summary of epigenetics and ageing, particularly in stem cell ageing, is reviewed in an independent section.

Together, the possible mechanisms discussed above help understand how chronic inflammation accumulate and persist during ageing. These factors also provide potential drug targets for therapeutic interventions to delay the ageing process and prevent inflammageing-associated diseases.

### Inflammageing-associated chronic diseases

**Fig. 4:**
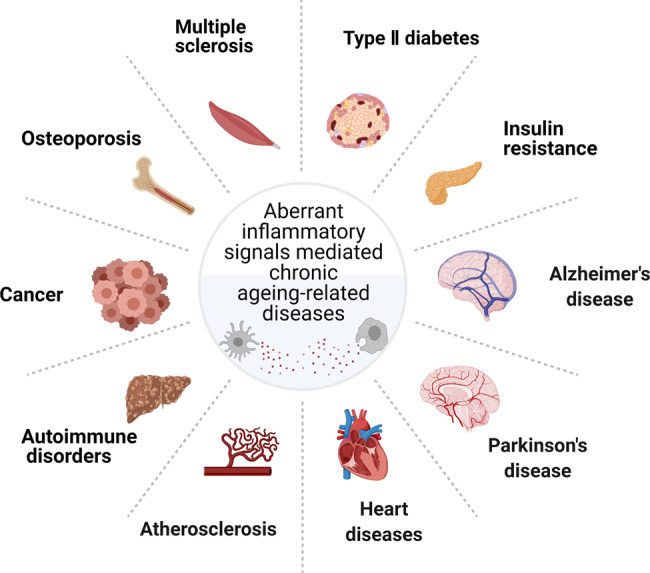
Inflammageing and its associated diseases. Multiple inflammatory signaling pathways, such as the NF-kB, NOTCH/JAG1, toll-like receptor, DNA damage response, cytosolic DNA sensing, autophagy, mRNA stabilization, and mTOR signaling pathways have been linked to age-related chronic diseases in various organs

Inflammatory signaling has beneficial functions in many physiological processes, such as embryo development and wound healing. However, excessive and persistent inflammatory responses are detrimental, as reflected by increased morbidity and mortality, leading to a decline in life quality. Indeed, several studies have shown experimental evidence linking inflammation to chronic age-related diseases^29^(Fig. 4). For instance, old mice were about 6.5-fold and fourfold more sensitive to the lethal toxicity of lipopolysaccharide and exogenous TNF than young controls, respectively.^128^ The enhanced sensitivity of old mice to inflammatory stimuli is possibly due to the already existing higher basal level of inflammation signal in aged animals. Therefore, the resolution phase is much more extended than young mice.^27,129^

It is worth noting that inflammation has been well established as a significant component of neurodegenerative disorders, yet it is unclear if this is a direct cause of the disease or a consequence of progressive neurodegeneration.^130,131^ Over the past decade, there has been a revolution in understanding how cytokines contribute to the etiology of the leading neurodegenerative disorders, including Alzheimer’s (AD) and Parkinson’s disease (PD). Inflammation also involves in many cardiovascular diseases, although whether inflammation causes a heart attack or other cardiovascular disorders require further investigation, inflammation serves as a universal sign for the atherogenic response. Atherosclerosis is a chronic inflammatory condition where atherosclerotic plaques show cellular senescence.^132,133^ Cytokines are involved in all stages of the pathogenesis of atherosclerosis, having both pro- or anti-atherogenic effects.^134,135^ Chronic tissue inflammation has a vital role in the etiology and immunopathogenesis of rheumatoid arthritis, with genetic and environmental factors contributing to a predilection to develop the disease.^136,137^ Osteoporosis is a disease in which bone loses calcium and become fragile. Young people maintain a balance between bone loss and bone formation. However, with ageing, the balance is disturbed toward bone loss due to the increases in chronic inflammation.^138^ Sustained low-grade inflammation can be found in type 2 diabetes due to a high concentration of circulatory inflammatory cytokines.^139^ The inflammageing also alters the function of the blood system, leading to decreases in lymphopoiesis, overproduction of myeloid cells, cytopenias, and anemia.^140–142^ Besides, the persistence of inflammageing intensively involves in hematological diseases, such as myelodysplastic syndrome and acute myelogenous leukemia.^143–145^ Other studies also link that inflammageing to hypertension, frailty, dementia, and chronic kidney disease.^146–148^

On the other hand, cell senescence inhibits aberrant cell proliferation and tumorigenesis, yet ageing is considered the most significant risk factor for cancer development. Nearly 60% of people suffering from various kinds of cancers are 65 years old or older. Paradoxically, although cellular senescence function as an anticancer program, the secreted SASP factors are associated with malignant tumor progression.^149^ The mechanism that links SASP and cancer have been extensively studied.^1^ For instance, IL-6 has been shown to activate WNT signaling and promotes cell proliferation.^150,151^ MPP3 (stromelysin) and vascular endothelial growth factor drive cancer cell invasion or tumor angiogenesis.^152,153^ Besides, SASP factors have been demonstrated to facilitate epithelial–mesenchymal transitions in the nearby premalignant epithelial cells, and resulted in cancer cell invasion and metastasis.^154,155^ Furthermore, many SASP factors can deteriorate the surroundings and remodel the tissue microenvironment, promoting cancer progression.^156–159^ Although the “seed and soil” theory has established for >100 years, the internal relationship between tumor microenvironment and cancer development becomes a research hotspot in recent years.^160^ The most abundant cells that compose the cancer microenvironment are cancer-associated fibroblasts (CAFs).^161,162^ Apart from the positive effect of CAFs on enhancing cancer proliferation and invasion, CAFs also contribute to tumor-associated inflammation.^163,164^ A study has shown that NF-kB signaling inhibition can abolish the effect of CAFs on promoting immune cell recruitment, neovascularization, and tumor growth in a mouse model of squamous skin carcinogenesis.^163^ Targeting on CAFs favors a positive role in extending life span and delaying cancer proliferation.^165^

### Therapeutic strategies to attenuate inflammageing

The pathways discussed above, which drive age-related inflammation, are potential therapeutic targets to modulate inflammageing and consequently, beneficial for the aged. One study indicates that switching off the immune machinery via mediating NLRP3 inflammasome activity could halt or even reverse these age-related diseases.^166^ Congruently, inhibition of NF-kB signaling could reduce the sensitivity of aged HSCs to inflammatory stimuli, leading to better maintenance of the hematopoietic system.^87^ Pharmaceutical or genetic removal of p16INK4a-positive senescent cells delays the ageing onset and tumorigenesis in the mouse model, although very recently p16^+^ liver sinusoid endothelial cells are found to be indispensable for the health span in mice.^7,167,168^ Furthermore, given the evidence that obesity causes increased inflammation, bodyweight control, and healthy diet consumption will be beneficial for reducing inflammageing.^61^ Similarly, exercise helps decrease inflammatory factors, which play an anti-inflammation effect across multiple systems, including cardiac, blood, and muscle.^75,169^ Likewise, CR lower inflammation and protect against age-related diseases. A recent study explored the effect of CR on multiple tissues at the single-cell level and found that genes related to immunity, inflammation, and lipid metabolism are most affected by the CR.^170^ Intake of reverse transcriptase inhibitor Lamivudine (3TC),^112^ or natural compounds represent a safe and effective option that helps ameliorate many age-associated disabilities and diseases.^93,112^ Resveratrol supplement could reduce ovarian inflammation, attenuated spinal cord injury, and suppressed tumorigenesis by targeting NF-kB and mTOR signaling in a SIRT1-dependent manner.^171–174^ Similarly, metformin supplementation can also reduce SASP by blocking NF-kB activity,^175^ although the sex-dependent effects on life span remain controversial upon metformin treatment.^176–180^ Other interventions, such as sleeping modulation, thymic replacement to increase adaptive immune function, maintain gut integrity, and to improve environmental quality, could be potentially helpful in altering the dynamic of inflammation and preventing the inflammageing related disease.^5^

Although these attempts have significant impacts on treating ageing and inflammation-associated diseases, the spatiotemporal regulation of pro-inflammatory cytokine release and its landscape have not been completely understood. Besides, due to the limited sensitivity of the current technique, many unknown age-associated pro-inflammatory cytokines in blood await to be detected. Nevertheless, single-cell omics and lineage tracing would surely empower a deeper understanding of inflammageing, and provide better solutions to counteract age-related inflammatory diseases.

## Ageing and epigenetics

Epigenetic regulation is used to classify heritable changes in gene expression that are not attributable to changes in DNA sequences.^181^ Mounting evidence suggests that epigenetic dysregulation is also an essential driver for cellular senescence and stem cell ageing.^182–186^ This part highlights the functional importance of epigenetic regulation in terminally differentiated cells and stem cells, in the context of altered DNA methylation, changes in histone modifications, and synergistic relationships between epigenetics and metabolism in ageing.

### DNA methylation

In mammalian cells, DNA methylation occurs predominantly at CpG dinucleotides. Methylated cytosine (mC) is found throughout the genome at high frequency, predominantly located at promoter regions of genes,^187^ which plays a critical role in transcriptional silencing.^188^ DNA methyltransferases (DNMTs) DNMT3A and DNMT3B establish genome-wide de novo methylated nucleotides, DNMT1 maintain methylated nucleotides, and TET protein family-regulated DNA demethylation.^187,189^

#### DNA methylation during cellular senescence

During cellular senescence, the landscapes of DNA methylation are changed in a context-dependent manner. For instance, local hypermethylation could be induced by senescence-associated heterochromatin foci (SAHF), which recruit DNMT1 to focal sites,^190,191^ while oncogene-induced senescence fails to exhibit such alterations in DNA methylation,^192^ reinforcing the diverse characteristics of DNA epigenetic alterations during senescence. Interestingly, mtDNA methylation has also been changed in replicative senescent cells. One study reveals that 76% of mtDNA noncoding regions are hypomethylated in senescent cells,^193^ where p53-induced downregulation of mitochondrial DNMT represents a possible mechanism for the hypomethylation of mtDNA.^194^ However, no significant global DNA methylation changes are observed in multiple forms of stress-induced premature senescence, including doxorubicin-induced senescence, irradiation-induced senescence, oncogene-induced senescence, and nonpermissive temperature-induced senescence.^195,196^ The different types of cell senescence-associated DNA methylation also lead to distinct gene expression patterns and cell phenotypes. Further investigations are required to explore the differences among the epigenetic mechanisms underlying replicative senescence and stress-induced premature senescence.

Of note, DNA methylation changes usually lead to a decline in the number and function of stem cells, like self-renew ability defect and differentiation bias, which are often similar to those observed in the ageing process.^1^ The effect of ageing on the DNA methylome of purified adult stem cells from young and old mice was detected by global methylated DNA immunoprecipitation sequencing.^197,198^ These studies show that HSCs display global DNA hypermethylation during ageing^197,198^ concomitant with decreased 5-hmC levels.^198^ Furthermore, ageing muscle stem cells (MuSCs) showed a slight increase in their DNA methylation age at the single-cell level.^199^

Ageing-associated gains of DNA methylation were also over-occupied at loci associated with polycomb gene (PcG) binding and some transcription factors binding in old HSCs.^197,198,200^ Similarly, PcG targets were also hypermethylated in MSCs during ageing, though a predominance of ageing-associated hypomethylation as reported.^201,202^ The correlation between age-related changes in DNA methylation and age-related changes in transcription was also examined in these studies, suggesting that the ageing process could disrupt these PcG proteins or transcription factors to bind DNA and regulate transcription.^201,202^

#### Sub-telomere region DNA methylation and ageing

Senescence-associated DNA methylation alterations are engaged in the regulation of telomere dysfunction. Telomere damage is not only determined by the telomere length, but also controlled by the epigenetic conditions in telomeric/sub-telomeric regions.^203^ In young wild-type cells, sub-telomeric regions are hypermethylation in CpG islands, and enriched by HP1a protein and repressive histone modification marks (H3K9me3 and H4K20me3), and lack of permissive histone modification marks (H3K9ac and H4K20ac).^203^ However, MEF cells from telomerase-deficient mice exhibit more “open” state of telomeric/sub-telomeric chromatin, as indicated by loss of CpG island DNA methylation, loss of repressive histone modifications (H3K9me3 and H4K20me3), decreased CBX3 binding accompanied by increased H3 and H4 acetylation, and increase the level of H3K9ac and H4K20ac.^203,204^

Our work discovered that the deletion of DDR factor Gadd45a rescued the heterochromatin remodeling via base excision repair-mediated active DNA demethylation in sub-telomeric regions in telomere-deficient cells, which generates an uncondensed chromatin structure to promote DDR signaling.^204^ Although Gadd45a has been linked to the global DNA methylation and transcriptional regulation, Gadd45a loss does not change the global DNA methylation pattern in our experimental setting,^204^ indicating that manipulation of the Gadd45a gene could delay organ ageing, and prolong the health span and life span of premature ageing mice.

#### DNA methylation and human ageing clocks

Age predictors based on a small set of CpG sites DNA methylation levels have been developed for humans and several other species.^205^ Early studies have found that age-related DNA hypomethylation patterns occur in many body tissues of the elderly.^187,205–209^ However, detailed analyses of several studies uncovered CpG islands site-specific DNA hypermethylation associated with ageing tissues,^200,206,210–213^ and these hypermethylation changes are generally related to age rather than the tissue type,^211,214,215^ suggesting some level of synergetic control of DNA methylome during ageing.

The DNA methylome of different organs or tissues can be used to predict the biological age.^216^ For example, DNA methylation in human peripheral blood has been manifested to correlate with ageing.^217,218^ A recent analysis of human blood samples does confirm that, with age, most hypermethylation is not related to changes in cell composition, but directly related to ageing.^213^ The increase in DNA methylation age of blood over 5 years was associated with a 16% higher mortality rate than age.^219,220^ Several research groups have observed an acceleration in DNA methylation age in some age-associated diseases, including AD, cardiovascular disease, and cancer.^221,222^ DNA methylation has been reported to regulate neuronal differentiation in early CNS development. A global methylome reconfiguration was associated with synaptogenesis ranging from mammalian fetal to adult brain development.^187^ In human, 353 CpG sites were identified to form an epigenetic age clock.^223^ The DNA methylation levels change with normal ageing in many tissues, including the brain, peripheral blood.^223^ Gene-specific DNA methylation changes are involved in rewarding in a context-dependent manner and are essential for memory formation, neurogenesis, and neuronal plasticity.^224,225^ Lower levels of DNA methylation on the promoter of target genes in peripheral blood samples have been reported to contribute to AD.^226,227^ The expression of DNMT1 and global 5mC and 5hmC were also shown to be decreased in AD neurons and hippocampus.^228,229^ Marioni et al.^230^ showed that greater DNA methylation acceleration is correlated with a lower cognitive score, weaker grip strength, and poorer lung function in humans during later life. Horvath et al.^231^ found accelerated DNA methylation age in Down syndrome patients with clinical signatures of “accelerated ageing.” There is also evidence that frailty, a syndrome with a pronounced association with age-related phenotypes, has a significant association with DNA methylation age, but not with telomere length.^232^ Zheng et al.^233^ pointed out that DNA methylation age estimated from blood tissue can also be used to predict cancer incidence and mortality. The apparent genetic clock derived from DNA methylation is better at estimating actual age than transcriptome and proteomic data or telomere length.^209^ In conclusion, the age-related DNA methylation changes may reflect the biological age to some extent, therefore constituting the biological age clock.

### Histone modifications

Histone modification is an additional epigenetic regulatory layer that is more complicated than DNA methylation. The unstructured N-terminal of histones can be used for posttranslational modification, including acetylation, methylation, phosphorylation, sumoylation, ubiquitination, and other modifications that change chromatin structure and accessibility. These modifications can regulate transcriptional activity. Here, we focus on histone acetylation and methylation, which are the two most well-studied markers in cellular senescence and ageing.

#### Histone (de-)acetylation and (de-)methylation during cellular senescence

Global decreases in H4K16Ac, H3K4me3, H3K9me3, and H3K27me3, while increases in the level of H3K9Ac and H4K20me3 occur in replicative senescent cells.^234–236^ Such histone modifications have also been found in stress-induced premature senescence cells.^237^ However, the histone marker alteration patterns differ in different stress-induced premature senescence cells based on various stress factors.^237^ Thus, the diversity of histone modifications in senescence cells may cause diverse gene expression patterns and senescence phenotype.

The role of senescence-associated histone modification changes in the senescence regulatory mechanisms has been broadly explored. The histone methyltransferases (HMTs) complex, polycomb repressive complex (PRC), was investigated in repressing the p16 gene expression.^238^ PRCs binds directly to the p16 locus and induces H3K27me3 occurrence, which leads to transcriptional suppression of p16.^239^ Besides, cell senescence can be delayed via inhibition of histone acetyltransferases (HATs) and induced by inhibiting histone deacetylases (HDACs).^240–242^ A recent study revealed that HAT p300 is a primary driver of the replicative senescence phenotype via a high-throughput screen.^242^ The depletion of p300 suppresses senescence-related gene expression, ensuing delayed senescence.^242^ Therefore, p300 is a candidate target for anti-ageing therapeutics.

However, certain histone modifications in senescence and ageing may be contrasting and even paradoxical. Tissue or cells from ageing organisms show increased H4K16ac, H4K20me3, or H3K4me3, along with decreased H3K9me3 and H3K27me3, which are quite different from cellular senescence.^243^ The difference in histone modification between cellular senescence and organismal ageing may attribute to multiple sources for ageing-associated damage, such as mutation, reactive oxygen species (ROS), and environmental stress, that change the epigenetic pattern of ageing and differ from cell senescence.^244,245^ In turn, epigenetic therapies based on histone modification that target cell senescence may inhibit the accumulation of senescent cells. Naturally occurring activators of SIRT1, including resveratrol, nicotinamide riboside, and nicotinamide mononucleotide, limit the accumulation of senescent cells.^246–249^

#### Histone acetylation and methylation during stem cells ageing

Histone associated epigenetic changes in adult stem cell ageing have been reported for numerous stem cell populations, remarkably HSCs and MuSCs.^125,250^ An increase in the level of the repressive histone modification H3K27me3 has been observed in both HSCs and MuSCs.^251^ However, H3K4me3, an active histone modification mark, shows an increase in HSCs but decreases marginally in MuSCs with age.^250^

Many genetic studies have revealed the critical role of HDACs and HATs activity in stem cell function. In the hematopoietic system, the significant phenotypes related to CREB-binding protein suggest the vital role of HATs in HSC function.^252–254^ Mononuclear leukemia zinc finger protein Moz is a kind of HATs translocation protein in human acute myeloid leukemia. During embryo development, the gene is eliminated, resulting in the severe loss of HSC and other progenitors with limited lineage.^255^ These results strongly suggest that histone acetylation is necessary for HSC self-renewal. HAT activity also plays an essential role in the homeostasis and function of HSCs and precursor cells. Eighteen mammalian HDACs have been identified and divided into four families. Class I HDAC plays a role in differentiation and seems to have a high degree of functional redundancy.^256,257^ In the hematopoietic system, loss of HDAC class I leads to a decrease in bone marrow cells,^258^ and in some cases, causes loss of stem cells and progenitor cells.^259^ In addition, the simultaneous knockout of HDAC3, HDAC5, and HDAC7 (class I and class II HDACs) resulted in the CDKN1A (p21) upregulation and inhibition of cell proliferation,^260^ similar to p21 induction and cell cycle arrest in human mesenchymal stem cells after drug-induced inhibition of HDAC activity.^261^

Class III HDAC includes the NAD^+^-dependent sirtuin family, while other HDAC families need Zn^2+^ as a cofactor. In mesenchymal stem cells, SIRT1 is related to differentiation into bone and cartilage by deacetylation of β-catenin.^262^ In adult neural stem cells, the loss of SIRT1 leads to increased self-renewal and proliferation with the increase of oligodendrocytes.^263^ Similarly, SIRT2 also hinders the differentiation of oligodendrocytes.^264^ In adult HSCs, loss of SIRT6 leads to enhanced Wnt signaling, decreased self-renewal, and over-proliferation.^265^ Similarly, SIRT1 guides the differentiation of epidermal stem cells by promoting the production of keratinocytes.^266^ In HSCs and MuSCs, SIRT1 loss leads to premature cell differentiation, implicating it is a regulator of self-renewal of these cells.^267–269^ Robust SIRT1 activity is also related to maintaining the quiescence of MuSCs, while the decrease of SIRT1 activity measured by increasing H4K16ac is related to the decline in NAD^+^ level in activated MuSCs.^267^ The increase of the H4K16ac level in these activated stem cells is due to the transformation of metabolism from fatty acid oxidation to glycolysis. Interestingly, although HSCs utilize glycolysis rather than oxidative phosphorylation (OXPHOS) and therefore have low levels of available NAD^+^, SIRT1 activity seems to be needed to regulate histone acetylation to maintain proper HSC function in ageing.^268^

The level of H4K16ac of aged HSCs was increased by immunostaining.^270^ Interestingly, compared with the young HSCs with high-level polarized H4K16ac expression, the H4K16ac level in the old HSCs decreased, concurrent with significantly changed cell distribution.^270^ The drug inhibition of Cdc42 and partial recovery HSC function reversed the change of H4K16ac in aged HSCs.^270^ Although the exact role of altered H4K16ac in aged HSC remains elusive, H4K16 deacetylation has been shown to hinder DDR and repair of double-strand breaks.^271^ Therefore, H4K16 deacetylation in aged HSC may contribute to the accumulation of DNA damage.^272^

In contrast to histone acetylation, histone methylation can be used as a context-dependent inhibitor or permissive marker, which indirectly regulates gene expression. Although histone methylation occurs on lysine and arginine residues, most stem cell studies detect methylation catalyzed by HMT on lysine residues.^251^ Changes in some other chromatin features, most of which are also altered with age, have been shown to regulate stem cell function.^273^ For instance, the H3K27me3 demethylase UTX is essential for MuSC-mediated muscle regeneration.^274^ The increase of histone inhibitory markers, such as H3k9me3 and H3K27me3, were observed in the aged MuSCs and HSCs, which indicated that the heterochromatin increased gradually in the ageing process.^251^ H3K4me3, an active chromatin marker, was enriched in the old HSCs, suggesting that epigenetic enhancement was observed in the transcriptional activation of stemness related genes.^198,251^ Contrary to what was observed in HSCs, the detection of H3k4me3 in MuSCs showed little difference between the cells isolated from young and old mice.^250^

Furthermore, H3K27me3 was added by polyclonal inhibition complex 2 (PRC2). When compared with the young HSCs, the level of H3K27me3 signals in aged HSC is essentially the same, but the coverage and intensity of H3K27me3 signals in aged HSC are expanded.^275^ However, unlike the young MuSCs, the aged MuSCs showed a transition to the euchromatin state after activation, with increased histone acetylation while decreased in H3K27me3.^250,276^ How the changes of H3K27me3 in the ageing process affect the function of stem cells in muscle and blood remain unanswered, yet this inhibitory marker may limit the regeneration potential of these stem cells, which will decrease with age.

### Epigenetic defects in progeroid laminopathies

The reconstruction of the chromosomal domain is also a feature of senescent cells. Senescent cells undergo substantial changes in three-dimensional chromatin organization globally, as evidenced by the combination of whole-genome chromosome conformation capture (Hi-C), fluorescence in situ hybridization, and in silico modeling.^277^ Among them, lamin A/C represents an epigenetic regulator of ageing partially due to its direct interaction with chromatin in a specific DNA sequence termed the lamin A-associated domains (LADs).^278,279^ In the process of cell senescence induced by an oncogene, the remodeling of LADs results in the unexpected recruitment of the decompressive sequence to the nuclear plate.^280,281^ Lamin A/C also promotes epigenetic changes by interacting with epigenetic enzymes.^282^ Under physiological conditions and in young cells, lamin A/C interacts with SIRT1 and enhances its deacetylase activity. It also promotes SIRT6 function during DNA repair and is found to recruit HDAC2.^283–285^ Importantly, the interaction of laminin with SIRT1, HDAC2, and SIRT6 decreased when protein A or progerin accumulated.^283,285^

The level of H3k9me3 in Hutchinson-Gilford progeria syndrome (HGPS) and mandibulofacial dysplasia type A (MADA) cells were decreased.^286,287^ HGPS cells lose heterochromatin protein HP1 and other heterochromatin markers, including H3K27me3 and H4K20me3.^287,288^ In addition, increased H4K16ac and H3K9ac were reported in HGPS cells and MADA cells, respectively.^285^ Congruently, many changes in DNA methylation levels at specific CpG sites were observed in the immortalized B cells of HGPS patients.^289^ Interestingly, according to the epigenetic clock,^231^ the same cells are older than expected. LADs were also involved in the epigenetic landscape remodeling of HGPS cells.^290^ In HGPS cells, progerin destroys the interaction of lamin A/C with SIRT1 and SIRT6, affecting chromatin localization and deacetylase function.^283,284^

### Epigenetic regulation of retrotransposable elements during ageing

Two subtypes of non-LTR retrotransposons, LINEs and SINEs, together make up nearly half of the human genome.^291–293^ Heterochromatin region in young cells and organisms silences reversible transposable factors, but due to the lack of regulation of higher-order chromatin structure, they are activated in the context of cell senescence and tissue ageing.^294^ Interventions to prolong life, such as CR, decreased retrotransposon expression in elderly mice.^295^ In the liver and muscle cells of old mice, CR delayed the loss of constitutive heterochromatin and inhibited the expression of repetitive components, including LINE1 and satellite components, which were in the centromeric, pericentromeric, and telomeric region. CR also inhibited the interaction between microRNA and chromodomain helicase DNA-binding protein 1, thus preventing the activation of retrotransposons induced by ageing and poor diet.^296^ Therefore, blocking the transcription of endogenous retrotransposon factors through diet restriction can improve the age-related phenotype, and support the view that retrotransposon leads to ageing and age-related diseases.

SIRT6 mono-ADP ribosylates KAP1 and promotes KAP1 interaction with HP1α, packaged as inhibitory heterochromatin in the LINE1 element. SIRT1 also binds to and inhibits major satellite repeats in yeast and mammalian cells.^297^ Another known heterochromatin regulator, retinoblastoma protein (Rb), antagonizes the activation of LINE1 in senescent cells and decreases the percentage of Rb on the LINE1 promoter in senescent human cells and senescent mouse tissues.^298^ Homologous protein transcription factors inhibit the expression of LINE1 in adult dopaminergic neurons^299^ and prevent its degeneration. In addition, partial overexpression of histone H3 and H4 reversed the transcription defects observed during ageing and reduced the reverse transcriptional transposition, which indicated that the increased reverse transcriptional transposition in old yeast was the result of histone loss during ageing.^291^ In addition, CRISPR-Cas9 screening also provided a genome-wide gene investigation related to the retrotransposition control of LINE1, revealing that the vertebrate-specific chromatin-modifying complex human silencing hub and the subunit of MORC family CW zinc finger protein 2 promoted the deposition of H3K9me3 to silence the transcription of LINE1 element.^300,301^ Recent studies have further shown that inhibition of the transposition of LINE1 by nucleoside reverse transcriptase inhibitors can inhibit the secretion of SASP and IFN by senescent human fibroblasts, and prolong the life span of the D. melanocyte model without DICR2, a heterochromatin structure regulating gene.^112^ These findings collectively suggest that epigenetic remodeling plays a vital role in the anti-ageing process, preventing the activation, and mobilization of retrotransposons by increasing heterochromatin stability. Genome-wide quantitative analysis will provide new insights into the frequency, structure, and location of retrotransposons during ageing, and clarify their overall contribution to ageing and rejuvenation.

### Sirtuin-mediated epigenetic regulation in stem cell ageing

Metabolism and epigenetics are closely linked, which together affect the ageing of the body. The availability of key nutrients, such as glucose, fatty acids, and amino acids, directly affects organisms’ life span. Glycolysis disorders have been shown to prolong life span^302^ and supplement D-glucosamine, an antagonist of glucose, which can damage glucose metabolism and prolong the life span of nematodes and mice.^303^ In addition, amino acid and lipid composition are closely related to age. They can be used as indicators of health span, as shown by the metabonomics analysis of plasma of healthy young and old individuals.^304^ However, mitochondrial metabolism is the most correlative with epigenetic regulation. Small molecules, such as NAD^+^, alpha-ketoglutarate (α-KG), and coenzyme A derived from mitochondria, have changes in the content of these metabolites during the ageing process and affect the activity of enzymes that use these metabolites as substrates for epi-modification.

The sirtuin protein family is one of the first known epigenetic enzymes and a key regulator of ageing and CR.^305,306^ In mammals, the sirtuin family contains seven Sir2 homologs, Sirt1–Sirt7, whose expression or enzyme activity increases after CR. It is worth noting that CR can prolong the life span of mice by inducing SIRT1 expression.^4^ Congruently, SIRT1 overexpression mimics the beneficial effects of CR. SIRT6 deficiency resulted in a shortened life span in mice and early death in nonhuman primates.^110,307^ In contrast, SIRT6 overexpression and CR induced SIRT6 activation delayed ageing.^282^ In addition, sirtuin activators, such as SRT1720 or SRT2140, can increase the health span of obese mice and the life span of mice on a standard diet.^308,309^ These longevity-extending effects of sirtuins are realized mainly by their enzyme functions, such as deacetylase and single ADP ribosyltransferase, especially when histone is used as the substrate. Recent findings of sirtuins in epigenetic regulation of adult stem cells are summarized below.

#### SIRT1

SIRT1 is very important to maintain the static and regeneration ability of HSCs under environmental stress and ageing conditions.^310^ Under normal circumstances, no abnormality was seen in the hematopoietic cells of SIRT1-KO mice.^311^ However, the hematopoietic commitment of SIRT1-deficient HSC is impaired in vitro, concurrent with a reduced survival rate of hematopoietic progenitor cells, especially in hypoxia or delayed addition of growth factor.^312^ Moreover, conditional ablation of SIRT1 in adult hematopoietic stem/progenitor cells (HSPCs) autonomously induces HSPCs expansion and loss of long-term repopulation under stress.^310^ This stress-induced loss of HSC function is associated with genomic instability, p53 activation, and increased DNA damage in SIRT1-deficient HSPCs.^310^ SIRT1 deficiency also resulted in a significant increase in H4K16ac and upregulated the expression of HOXA9, a key regulator of HSPC function and proliferation.^268,310,312^ In general, SIRT1 is essential to maintain different ASC pools by maintaining quiescence, self-renewal, and regenerative capacity, especially in response to stress and injury.

#### SIRT2

Sirt2 is a mammalian sirtuin, which primarily exists in the cytosol and has deacetylase activity.^313^ Luo et al. suggested that mitochondrial stress and SIRT2 inactivation lead to the activation of inflammatory corpuscles in NLRP3 of HSC and the decline of HSC ageing function.^48^ Specifically, diminished SIRT2 expression augmented mitochondrial stress, while SIRT2 overexpression or NLRP3/caspase-1 inactivation attenuated the impaired regenerative capacity of aged HSCs.^48^

#### Mitochondrial sirtuins (Sirt3, 4, and 5)

In addition to the three nuclear sirtuins (Sirt1, 6, and 7), mitochondrial sirtuins (mtSIRT) include three members, i.e., SIRT3, SIRT4, and SIRT5, which are all involved in regulating stem cell metabolism.^314^ Among them, mitochondrial SIRT3 was found to be highly enriched in HSCs.^315^ Brown et al. suggested that SIRT3 deficiency reduced the HSC pool of old mice and impaired the self-renewal of HSC during continuous transplantation stress, partly due to the hyperacetylation of superoxide dismutase (SOD2) and subsequent increase in oxidative stress.^315^ SIRT3 decreased with age and its overexpression in HSC reduced oxidative stress and maintained reconstructive ability.^315^ SIRT4 expression is upregulated during cell senescence of different types.^316^ SIRT4 overexpression can induce trophoblast stem cell senescence.^317^ SIRT5 has demalonylase, deglutarylase, and desuccinylase activities^318,319^ that regulates ammonia detoxification.^320^ Although not well studied in stem cells, SIRT5 was thought to desuccinylate and activate SOD1 to maintain a low ROS level in stem cells.^321^

#### SIRT6

Specific deletion of Sirt6 in HSCs of adult mice resulted in the amplification of HSPC, which is associated with acetylation of H3K56 and the increase of transcriptional factor in the Wnt signaling pathway.^265^ The increased proliferation further hampered HSC quiescence and led to HSC depletion. As a result, the long-term regeneration capacity of Sirt6-defective HSCs was severely impaired.^265^ Furthermore, Sirt6-mediated stress resistance also helps maintain the in vitro function of MSCs.^322^ Human bone marrow mesenchymal stem cells derived from SIRT6-deficient mice were susceptible to oxidative damage due to the increased level of ROS.^322^ Mechanistically, the SIRT6-mediated antioxidant effect by H3K56ac deacetylation activated the Nrf2-mediated antioxidant gene.^322^ Although it is not clear how the deacetylase acts as a coactivator to promote anti-oxidation genes in MSC, this study shows that SIRT6 is crucial to mediate the anti-stress and anti-ageing effect of MSC.

#### SIRT7

Sirt7 is also highly expressed in HSC, which exerts its regenerative ability by regulating the unfolded protein response in mitochondria.^323^ Quiescent HSCs are maintained in a state of inactive metabolism that can be easily activated via boosting mitochondrial content. Mohrin et al. showed that mitochondrial protein folding stress (PFSmt) induced the interaction between Sirt7 and Nrf1, and inhibited the expression of mitochondrial ribosomal protein and mitochondrial translation factor. Sirt7-deficient HSCs exited quiescence and exhibited an ageing phenotype, including the increase of PFSmt, apoptosis, a decrease of reproductivity, and biased bone marrow differentiation. On the contrary, the upregulation of Sirt7 improved the regenerative ability of HSC in the elderly.^323^

Together, posttranscriptional and posttranslational regulation via sirtuins maintains stem cell function to cope with various stress stimuli and ageing. Although mtSIRT is less studied in stem cell function, three nuclear sirtuins (Sirt1, 6, and 7) participate in the static control of stem cells, which is of considerable significance to maintain the regenerative ability of stem cells and prevent premature ageing. Pharmacological interventions targeting sirtuins may hold great promises to counteract stem cell ageing and, therefore, tissue homeostasis.

### Approaches in epigenetic control of rejuvenation

Somatic cells are induced to restore pluripotency through various reprogramming strategies, the most common of which is the overexpression of four transcription factors Oct4, Sox2, Klf4, and Myc (referred to as OSKM).^324^ The mouse nucleus reprogrammed by OSKM can produce viable embryos and further develop into fertile adults without showing premature ageing, indicating that the time sequence of the donor nucleus has been reset. Consequently, core reprogramming appears to be able to reset ageing clocks. Partial reprogramming of the adeno-associated virus vector expressing OSKM can significantly improve axon regeneration after injury.^325^ The expression of OSKM leads to extensive remodeling of chromatin, accompanied by alterations of epigenetic enzymes and other transcription factors. For example, as a pioneer factor, Oct4 can loosen heterochromatin and reduce the global levels of inhibitory H3K9me2, H3K9me3, and 5-methylcytosine,^326^ which are obstacles in reprogramming.^327,328^ In addition, during OSKM-mediated pluripotent reprogramming, the epigenetic memory of the primitive cells is largely eliminated and rewritten in the subsequent differentiation process.^329^

Many drugs, compounds, and supplements with anti-ageing properties have also been identified and attracted considerable attention by pharmaceuticals, which can prolong the life span and healthy ageing of model organisms (such as mice, *Drosophila melanogaster*, and *Caenorhabditis* *elegans*).^330^ For instance, metformin regulates the activation of AMPK, which directly governs the activities of several epigenetic enzymes, such as HATs, HDACs, and DNMTs.^331,332^ In addition, metformin restores AMPK-mediated phosphorylation and stabilizes Tet2, thereby preventing changes in 5-hydroxymethylcytosine levels.^333^ Aspirin supplementation has also been shown to generalize the anti-ageing effect of CR.^334^ The accumulation of senescent cells is one of the signs of ageing. Senolytics selectively eliminate senescent cells, representing a new anti-ageing drug^335^ that may delay the ageing process. Consistently, eliminating p16INK4a-positive senescent cells was able to prolong the life span of early ageing model mice and wild-type mice.^167,336^

Metabolic intermediates and by-products of the tricarboxylic acid cycle act as cofactors and substrates of various epigenetic enzymes, including acetyl CoA for acetylation and S-adenosylmethionine (SAM) for methylation.^337,338^ In addition, α-KG, an intermediate product of the tricarboxylic acid cycle, induces DNA and histone demethylation by activating the jumoniji C domain-containing demethylase and lysine demethylase. A recent study showed that the increase of α-KG activated JMJD3 (histone H3K27 demethylase) and PHF8 (histone lysine demethylase H3K9me1/2 specificity), leading to the removal of inhibitory markers and the induction of mitochondrial unfolded protein response gene expression. These changes are enough to prolong the life span of nematodes.^339,340^

Another important metabolite is NAD^+^, which is the cofactor of sirtuins. It connects the gene regulation of epigenetic with mitochondria. High levels of NAD^+^ can improve mitochondrial function, supplement the stem cell pool, and prolong the life span in mice. Supplementation of NAD^+^ precursor to aged mice can delay the decline of mitochondrial function, improve muscle, nerve, and melanocyte stem cell performance, alleviate age-related physiological decline (for example, type 2 diabetes and cognitive impairment), and prolong life span.^341^

Apart from the investigations of novel epigenetic interventions in pursuit of rejuvenation, the study of epigenetic regulation at the single-cell resolution has deepened our understanding of the diversity of the single-cell state and the process of cell state maintenance.^342–344^ Specific single-cell DNA sequencing provides epigenetic information of DNA modification, DNA accessibility, and chromosome conformation, thus deepening the understanding of the impact of epigenome on the transcriptome. A variety of single-cell epigenome-sequencing techniques have been developed, such as single-cell sodium bisulfite sequencing for DNA methylation detection,^345^ single-cell chromatin immunoprecipitation sequencing for the identification of histone modification and protein–DNA interaction, single-cell transposable accessible chromatin sequencing, and Hi-C sequencing for the evaluation of chromatin accessibility and chromosome conformational information.^346–348^ These techniques have been combined with single-cell transcriptome to study gene regulatory profiles and analyze cell heterogeneity. Recently, a new method has been established to record transcriptome synchronized with chromatin accessibility, thus enabling the analyses of the functional relationship between these two characteristics in the same cell.^349^

The intensity of the regulatory link between epigenetic modification and transcription may vary in different developmental stages and cell types, adding a layer of complexity and uncertainty in delineating the specific spatiotemporal transcriptional regulation. The application of these single-cell and multi-omics techniques enables us to understand the regulation of epigenetic factors on gene expression under physiological and pathological conditions.^350^

**Fig. 5:**
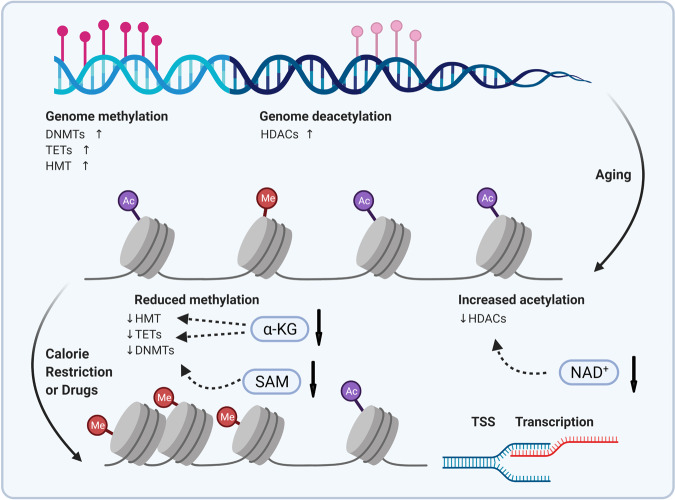
Epigenetic regulators and interventions in ageing. Age-dependent epigenetic remodeling by various (de-)methylases and (de-)acetylases affects chromatin accessibility, thereby regulating gene transcription and expression. Interventions, such as calorie restriction and drug treatment, may reverse the age-dependent degeneration in an epigenetic-modifying manner. HDACs histone deacetylases, DNMTs DNA methyltransferases, TET ten–eleven translocation, HMT histone methyltransferase, TSS transcriptional start site, SAM S-adenosylmethionine, α-KG alpha-ketoglutarate, NAD nicotinamide adenine dinucleotide

In sum, stem cells residing in different tissues accumulate defects during ageing, preventing stem cells from repairing damage, and maintaining tissue homeostasis, with altered epigenetics a potential hallmark (Fig. 5). Existing evidence has suggested that the developmental pathway in embryogenesis is the key to epigenetic regulation that contributes to stem cell ageing. These observations raise important issues for future research. While it is unclear whether epigenomic changes are an essential element of ageing and how these changes occur during ageing remain elusive, many attempts are on the way to depict the full landscape of acute and chronic changes in the epigenetic modification during stem cell ageing. Given that mutations in epigenetic modifiers have become a marker of the ageing hematopoietic system, understanding the ageing-related clonal dominance mechanism of stem cells with mutations in epigenetic modifiers is of great interest. Finally, epigenetic integration of damage signals, as a hardly neglected cause of stem cell and organismal ageing, has brought new hope for the translational pathway. Since the epigenome changes are largely reversible in principle, manipulating epigenetic imprints holds great prospects in improving tissue maintenance, regeneration ability, and, ultimately, extending health span.

## Ageing and metabolism

**Fig. 6:**
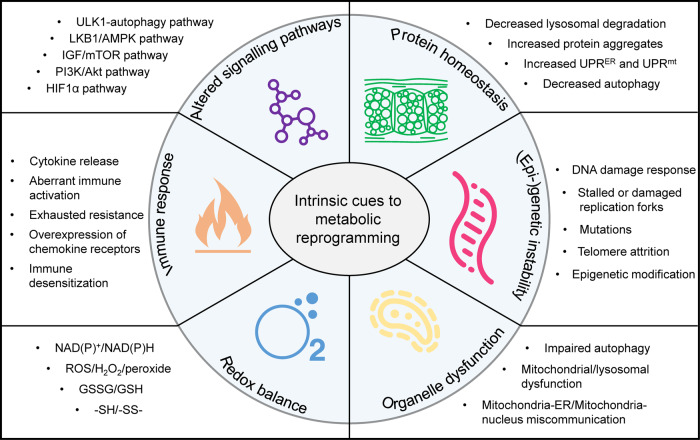
Intrinsic cues to metabolic reprogramming during ageing. Six aspects, namely protein homeostasis, genetic and epigenetic instability, organelle dysfunction, redox imbalance, immune response, and altered signaling pathways, lead to the metabolic regulation of ageing

The unique metabolic signature of senescent cells shapes the distinct senescent phenotype; for instance, augmented glycolysis represents one of the metabolic hallmarks during replicative senescence. Changes in intracellular and extracellular metabolites may lead to the consequence of senescence in adjacent cells, aka a bystander effect of the senescence-associated metabolic pattern.^351^ This section will discuss the metabolic regulation of cell senescence that interacts with many aspects of cellular physiology, including redox balance, genomic integrity, immunometabolism, proteostasis, organelle homeostasis, and metabolic signaling pathways and interventions (Fig. 6).

### Metabolic regulation of redox balance in ageing

Redox reactions occur throughout the cellular metabolism with the production of a small number of reactive oxygen radicals.^352^ Studies using model organisms, such as yeast, nematode, drosophila, and mouse have shown that cell senescence is closely related to dysregulation of redox balance.^353–356^ With ageing, increased oxidative stress featured by increased oxidized glutathione (GSSG), while lowered levels of glutathione (GSH) and reduced form of nicotinamide adenine dinucleotide phosphate (NADPH) have been observed, which causes lipid, protein, and DNA damages (e.g., DNA single- or double-stranded breaks, fatty acid chain breakage, increased membrane fluidity, and protein hydrolysis, inactivation of proteases, etc.). All of the above injuries can lead to aberrant cellular metabolism and signal transduction, culminating by altering cell fate.^357^ Consequently, systemic clearance of the excess free radicals through enzyme-based, such as SOD2, catalase, glutathione peroxidase, coenzyme Q10, etc.), and nonenzyme-based (e.g., vitamins, β-carotene, selenium, GSH/GSSG, cysteine, etc.) defense systems are required to maintain physiological balance. It has been generally accepted that redox imbalance caused by boosting of ROS and reactive nitrogen species (RNS) production, or “oxidative stress”, and elevated intracellular NADH:NAD^+^ ratio, or “reductive stress”, reduced OXPHOS lead to the disorders of the mitochondrial electron transport chain (ETC), therefore, decreased ATP synthesis and cell respiration.^358^

ATP synthesis utilizing various substrates is fundamental to cell viability. NAD^+^/NADH and FAD/FADH2 redox couples synthesize most ATP; in particular, NAD^+^ involves regulating both redox and metabolic homeostasis.^359^ Accumulation of mitochondrial proton donors (NADH and FADH2) attenuates OXPHOS through the ETC, thereby causing reductive stress and increased ROS production. ROS exerts multiple functions in various pathophysiological responses closely related to augmented NADH/NAD^+^ ratio and accumulation of L-2-hydroxyglutarate, a reductive metabolite that buffers reductive stress via inhibiting glycolysis and the Kreb’s cycle.^360^ Increased intracellular NADH concentration caused by hypoxia will generate a reverse electron transfer, resulting in higher succinate levels and increasing oxidation, and subsequently higher ROS levels.^360^ A similar process has been seen in the mechanically overloaded heart. Thus, a stable ratio of NAD^+^/NADH is essential to maintain cellular homeostasis. Any alteration in the ratio of NAD^+^/NADH will cause oxidative or reductive stress, which may lead to accelerated cell senescence. Accordingly, restoration of NAD^+^ via precursors may reestablish the NADH/NAD^+^ ratio, thereby reducing the cardiovascular injuries and attenuating cell senescence.^361–363^ Indeed, mounting studies have shown the influence of exogenous NAD^+^ repletion in the regulation and homeostasis in different models (gut, heart, muscle, etc.).^364–369^ A recent study shows augmented circulating α-hydroxybutyrate levels associated with increased NADH/NAD^+^ ratio and impaired glucose metabolism,^370^ while a normalized NADH/NAD^+^ ratio can achieve by constructing LOXCAT-mediated conversion of lactic acid to pyruvate.^371^

Although targeted regulation of the NADH/NAD^+^ ratio or NAD^+^ level has great potential as an intervention for cell senescence and organ ageing,^372^ there are still some critical questions about the relationship between NADH/NAD^+^ and cell senescence. For instance, in vivo NADH/NAD^+^ ratio and NAD^+^ level (particularly in different subcellular organelles) cannot be determined by conventional biochemical analysis. The exact mechanisms by which exogenous NAD^+^ and NADH work remain elusive. Furthermore, under the condition of ageing-related metabolic remodeling, the spatiotemporal distribution of NADH/NAD^+^ or NAD^+^ in different cellular compartments is still unclear. Given the crucial roles of NADH/NAD^+^ and NAD^+^ in metabolic regulations of redox balance and ageing, new technologies are urgently needed to provide a landscape of precise dynamics of NADH/NAD^+^ or NAD^+^ at subcellular organelle, cellular, and tissue levels. Some attempts have successfully detected NADH/NAD^+^ ratio or NAD^+^ concentration in vivo by constructing genetically encoded fluorescent probes and reporter mice,^373,374^ which hold high potentials to delineate the compartmentalized distribution of NADH/NAD^+^ ratio or NAD^+^ concentration upon various ageing-related stress stimuli.

Another vital player in redox balancing is the pentose phosphate pathway (PPP). PPP in the cytoplasm converts glucose into 5-phosphate ribose and produces NADPH.^375^ The reductive NADPH functions as an antioxidative mechanism produced during the oxidation phase, starting with the conversion of glucose-6-phosphate to 6-gluconolactone and NADPH. This is an irreversible reaction, catalyzed by glucose-6-phosphate dehydrogenase (G6PDH, a rate-limiting enzyme of this pathway), while inhibited by NADPH feedback. Next, the lactone hydrolyzes to 6-phosphogluconic acid that is further dehydrodecarboxylated to form ribulose 5-phosphate, concomitant with another molecule of NADPH. NADPH is used for the reductive reactions in the synthesis of biomolecules, such as fatty acids, cholesterol, deoxyribose, tetrahydrofolic acid, etc. NADPH is also used to reduce GSSG, thus maintaining redox balance in cells.^375^ The flux of PPP is mainly determined by the amount of reduction force. Besides, high ROS levels in senescent cells also require more NADPH to maintain redox balance.^376^

The causal role of oxidative damage in the ageing process remains controversial, partly because of the absence of a clear correlation between the efficacy of antioxidant defenses and extended cellular function or longevity.^377^ The lack of human studies on toxic oxidative metabolites in tissue ageing also makes scientists tiptoe cautiously at the crossroad. Nevertheless, existing evidence demonstrates that ageing-related pathological fibrosis can be attenuated via the Nox-4–Nrf2 antioxidant axis.^378^ Other interventions that promote cysteine metabolism and hydrogen sulfide production also exhibit therapeutic benefits and delay tissue ageing.^357,379^ Mitochondria represent the central platform of cellular metabolism and contain their own genome. The accumulation of mutations in mtDNA during ageing has been validated in many cell systems,^380^ with ROS as a principal cause.^381^ Despite several studies implicating the role of ROS in cell senescence, others also suggest that it may not necessarily be the case. One study using an empirical mathematical model (stochastic step model of replicative senescence) suggests that increased mitochondrial ROS production in replicative senescent cells is a consequence of the senescence phenotype rather than the reverse.^382^ Another report shows that overexpression of the mitochondrial localized antioxidant SOD2 and the mitochondrially targeted catalase are insufficient to inhibit the senescence phenotype in hyperoxia-induced senescent cells.^383^ Because mitochondrial and non-mitochondrial enzymes produce ROS during hyperoxia (70% O_2_), the inability of mitochondrial antioxidants to reverse growth arrest in hyperoxia-induced senescence suggests that cytosolic ROS may assist growth arrest. Hence, the mechanisms involved in linking mitochondrial ROS and cell senescence still need to be further studied. Nonetheless, studies on genetically manipulated mouse model suggested that many metabolic pathways have been found to involve oxidative stress management, and thus regulating cell function and maintenance, including the Akt/mTOR,^384^ FoxO,^385^ AMPK,^386,387^ the ATM-BID,^388^ Nrf2/Keap1,^389^ and the sirtuins,^315^ and so on. Together, these studies suggest that cell senescence is closely correlated with the metabolic and redox state, influencing the intracellular homeostasis of ROS and its functionalities.

### Metabolic regulation of DNA damage in ageing

Cells have evolved various systems to regulate nutrient availability to maintain homeostasis, and have also developed active DNA repair machinery to avoid detrimental genomic instability during ageing, with two distinct cellular activities highly coordinated.^102,390^ For example, premature ageing phenotypes in excision repair cross-complementing group 1 knockout mice and progeroid Xpg mice can be attenuated after feeding a restricted diet.^391^ During the neonatal, a high oxygen environment induces cardiomyocyte cell cycle arrest through DDR and directs perinatal cardiac metabolic switch.^392,393^ To date, many key regulatory molecules exerting dual roles in regulating DNA repair and cellular metabolism have been identified, including p53,^394^ sirtuins,^395,396^ poly(ADP-ribose) polymerases (PARPs),^397^ and ATM.^398^ All these factors converge to the telomere at the end of the chromosome in eukaryotes. Of note, telomere shortening represents one of the common mechanisms for organ ageing.^399^ Telomerase inactivation in early life accelerates ageing phenotypes regardless of telomere length.^400^ Gene mutations in the telomerase complex cause accelerated telomere erosions, which leads to heritable syndromes in multiple systems, takes congenital keratosis, for example, the main cause of death is DNA damage-related bone marrow failure.^401^ Consistently, telomerase gene mutations and accelerated telomere shortening have also been found in patients with aplastic anemia, suggesting that telomere shortening is closely related to the decline in adult stem cell regeneration, and premature ageing of tissues and organs.^402^ In high-turnover tissues, such as the hematopoietic system, we also found that telomere shortening triggers cell senescence or apoptosis by activating DDR,^403–405^ suggesting a close connection between DDR and telomere maintenance. Intriguingly, other studies have shown that telomere-related proteins can be localized on mitochondria, thus affecting mitochondrial metabolism.^406^ For instance, in low-turnover tissues, such as the heart, telomerase dysfunction perturbs cellular energy metabolism thus promoting ageing. The seminal study from DePinho’s lab demonstrated a direct link between telomere dysfunction-induced DDR to mitochondrial metabolic compromise, where critical shortening of telomere length induces p53 activation, thereby lowering PGC1 expression.^407^ Recently, another study also indicates that telomere attrition can lead to p53-dependent sirtuin repression,^408^ in turn triggering a metabolic remodeling. Since telomere attrition occurs in each cell division, at some time point, it will trigger a DDR (e.g., through PARP and ATM), thereby reducing the stability of the genome and eventually leading to cell senescence. Congruently, mice with hyper-long telomeres show less metabolic ageing and longer life spans.^409^ We also demonstrated that mild elevation of mitochondrial biogenesis regulator PGC1α in late-generation telomerase-deficient mice was sufficient to attenuate metabolic compromise and extending health span.^362^

#### p53

p53 represents a sophisticated molecule that wires the nuclear-metabolic axis during ageing; that is, p53 involves glycolysis, OXPHOS, and regulation of the PPP and other pathways via regulating a plethora of target genes.^410^ Under the ageing-related pseudo-hypoxic state, a reduced glycolytic rate can be achieved through the downregulation of monocarboxylate transporter 1 expression by p53-mediated prevention of lactate efflux.^411^ In addition, p53 activates the transcription of cytochrome *c* oxidase 2 synthesis, a core component of OXPHOS, and plays a key role in the regulation of cytochrome *c* oxidase complex assembly.^412^

p53 affects several physiological and metabolic pathways, all heavily involved in modulating ageing and the establishment of senescence.^413^ There is growing interest in dissecting how metabolism affects DDR and vice versa. One hallmark of aged cells is the deregulation of genes involving in DDR. The cell cycle, as well as senescence, can be regulated by metabolism and mitochondrial activity through p53. For instance, vascular smooth muscle cell senescence-induced atherosclerosis was associated with p53-dependent degradation of telomere repeat-binding factor-2.^414^ Besides, mitochondrial stress in the form of increased ROS levels also induces cell cycle arrest using mediators, such as p53 and p27.^415^ Meanwhile, phosphorylation of Ser-15 by ATM activates p53 in response to DNA damage.^416^ Our study also reveals a mechanism of cell senescence, in which distinct effects of p53 and mTORC1 pathways on HSC ageing are governed by Wild-type p53-induced phosphatase 1 (Wip1), which negatively regulates several tumor suppressors and DDR pathways,^417^ but also B lymphocyte maturation and tissue regeneration.^418,419^

#### ATM

First identified in 1995, the ATM kinase contains a PI3K-like kinase domain at the C-terminal and represents the core regulator for DNA double-strand break and repair. During ageing, ATM/ATR-dependent DDR, rather than the p53/p16INK4a axis, was required for the GATA-binding protein 4-mediated senescence-associate secretory phenotype.^420^ Cells isolated from AT patients were sensitive to radiation, indicating that ATM involves DNA repair. ATM is a relatively upstream kinase that phosphorylates hundreds of substrates in response to DNA damage. The MRE11–RAD50–NBS1 complex, Tip60, ATR, protein phosphatase 2A, and Wip1 regulate ATM activation. In turn, the activated ATM can activate checkpoint kinase 2, structural maintenance of chromosomes-1, Fanconi anemia, complementation group D2, breast cancer 1, early onset, and H2A.X variant histone. Studies have shown that oxidative stress can also activate ATM by phosphorylating ATM at Ser1981, resulting in phosphorylation of p53 and AMPKα, therefore regulating gene expression and energy metabolism. ATM deficiency in mice causes insulin resistance, increased adiposity, atherosclerosis, and a variety of metabolic syndromes,^421^ while ATM activation is required in insulin-induced glucose transport in slow and fast muscle fibers utilizing glucose oxidation.^422^ Intriguingly, the repletion of NAD also attenuated the severity of AT-induced neuropathology, suggesting that accumulated DNA damage links to dysfunctional mitochondrial metabolism.^423^ ATM is the sensor for ROS in human fibroblasts, which also mediates mitochondrial ROS signaling and extends the life span of yeast. A more recent study demonstrated that ATM promotes antioxidant defenses and the repair of double-strand DNA breaks by activating the PPP, representing a link between DNA repair processes and cellular metabolism.

#### Sirtuins

Sirtuins are a cluster of NAD^+^-dependent enzymes involving the regulation of metabolism and genome integrity. Many proteins related to DNA repair have been identified as direct substrates of sirtuins, e.g., RB-binding protein 8, endonuclease (CtIP), PARPs, and p53.^424–426^ Conversely, p53 can posttranscriptionally regulate non-mtSIRT via miRNAs, while transcriptionally regulating mtSIRT through PGC1α/β.^408^ Existing evidence manifests that nuclear DNA damage signal to the mitochondria during CR with age, where sirtuin network bridges such nucleus–mitochondria communication linking genome instability to altered mitochondrial metabolism.^427^ Another evidence shows that sirt3 deacetylates and activates IDH2 to boost NADPH, thus mediating reduced oxidative stress and DNA damage.^428^ In addition, sirt7 deacetylates p53 to inactivate its pro-apoptotic effect, thereby attenuating cardiac hypertrophy and inflammatory cardiomyopathy.^429^ These observations lead to attempts to boost sirtuin as an anti-ageing strategy. Indeed, sirtuin overexpression exerts a salutary effect in healthy ageing by enhancing the resistance to DNA damage and metabolic insult.^430^

#### PARP

PARP represents another NAD^+^-consuming enzyme that catalyzes the formation of PAR polymers. As a base excision repair protein, PARP overactivation has been linked to mitochondrial dysfunction in xeroderma pigmentosum group A, as well as ataxia telangiectasia and Cockayne syndrome with similar pathogenesis involving nuclear–mitochondrial communication.^426^ Prolonged PARP activation also couples NAD^+^ and ATP depletion, thereby triggering cellular energy crisis and metabolic reprogramming.^431,432^ Some metabolic transcriptional factors (e.g., HIF-1a, NRF-1, and NRF-2) and nuclear receptors (e.g., estrogen receptor, retinoid X receptor, and peroxisome proliferator-activated receptors) have been identified to interact with PARP, affecting glucose and lipid metabolism and related metabolic syndromes.^433^ Collectively, these data suggest a link between DNA damage-induced poly(ADP-ribosyl)ation and mammalian longevity.^434^

In sum, the accumulation of DNA damage can promote metabolic dysfunction in two ways: cell-autonomous and non-cell-autonomous mechanisms. Tissue regeneration is impaired by DNA damage-induced senescence and/or apoptosis of stem cells and somatic cells, leading to metabolic dysfunction. DNA damage induces non-cell-autonomous tissue inflammation through the upregulation of cytokines and chemokines, thereby hampering systemic insulin signaling. DNA damage can also affect systemic metabolic homeostasis through influences on cellular metabolism and the endocrine system. Activation of the DDR in specific tissues could influence the function of vital metabolic organs, thereby provoking systemic insulin resistance. Thus, a better understanding of the systemic DDR may help us develop novel therapeutic strategies for metabolic disorders. Additional factors await identification to shed further light on the role of DNA damage in metabolic homeostasis.

### Metabolic regulation of immune response in ageing

Inflammation is one of the most described ageing-related phenotypes in ageing research. Senescent cells activate innate and adaptive immune responses that can be beneficial and detrimental.^435^ Meanwhile, immune organs senesce rapidly after advanced age, causing diseases such as infection, cancer, and other many chronic inflammatory diseases.^436,437^ In response to ageing-related intrinsic and extrinsic factors, induction into pro-inflammatory cell senescence represents a conserved antitumor and pro-survival mechanism that promotes the clearance of damaged components, restriction of fibrosis and tissue repair, thereby maintaining the relative homeostasis.^438^ However, persistently activated immunosurveillance can accelerate immune senescence, featured by a blunted immune response, bias to myeloid hematopoiesis, and impaired immunometabolism, possibly stem from the ageing of haematopoietic stem cells.^403,439^ Many investigations reveal that different stimuli can lead to age-related inflammatory manifestations, such as DDRs, telomere attrition, oncogene activation, oxidative stress, chemotherapy drugs, or radiation damage, all of which contribute to the SASP.^440–443^ In turn, SASP causes an inflammatory microenvironment that dampens tissue regeneration.^444^ Notably, the metabolic signatures of senescent cells have been intensively investigated, with some typical changes in immunometabolism during ageing discussed below.

#### Altered secretome

The altered secretory phenotype in inflammatory cell senescence includes (1) altered nucleic acid secretion, such as micronucleus formation and disruption, leakage of mtDNA, release of LINE1, which may activate RIG-I-MAVS or cGAS–STING signaling pathways to initiate an inflammatory response;^110,445^ (2) altered protein secretion, such as inflammatory chemokines, cytokines, and other factors, which may exert tissue repairing or degrading effect to eliminate danger signals;^446^ (3) altered metabolite secretion, such as carnitine and low density lipoprotein, which may correlate with risk of ageing-related oncogenic diseases, cardiovascular, and cerebrovascular diseases.^447^ Of note, such DAMPs can activate the cellular immune response that differs from the pro-inflammatory response of immune cells induced by pathogens (PAMP), indicating the secretome signature to be specific and stimulus-dependent.^448^ Moreover, energy demand changes dramatically when immune cells shift from a resting state to an active state, which often causes energy deficits when cells become senescent and, consequently, contributes to the metabolic remodeling accompanied by the altered secretion of cytokines, chemokines, and inflammatory mediators.

#### Extracellular vesicle secretion

Extracellular vesicles (EVs) are bilayer vesicles released from cells into the extracellular matrix.^449^ For many years, EVs have been regarded as a mechanism for excreting intracellular components as waste products, and are important mediators for exchanging proteins and lipids between secretory cells and target cells. Two major types of EVs, microvesicles, and exosomes contain a variety of components (e.g., enzymes, miRNAs, transcription factors, membrane-bound and soluble receptors, lipids, and DNA, etc.) and involve in cell communication, migration, angiogenesis, and tumor cell growth. Mounting evidence has been suggested that EV secretion plays a crucial role in age-related inflammatory and metabolic abnormalities.^450^ For instance, one study demonstrates that the levels of extracellular nicotinamide phosphoribosyltransferase (eNAMPT) decline with age in mice and humans. Increasing eNAMPT promotes NAD^+^, counteracting ageing, and extending health span.^451^ Other studies reveal that EVs participate in metabolic reprogramming during macrophage polarization,^452^ wound healing,^453^ and fibrotic replacement.^454,455^ EVs derived from immune cells contain antigen peptide–MHC complex and various antigens, which can control the exchange of antigen information between immune cells, thus regulating activation or inhibition of immune cells and other immune responses.

#### Metabolic reprogramming

Throughout the life of an organism, the immune system continually senses and responds to environmental threats, which is a relatively energy-consuming process.^456^ Altered energy substrate (e.g., glucose, lipid, and glutamate) metabolism is commonly found in the stressed cells.^339^ Metabolic pathways control the duration and strength of innate and adaptive immune responses and the generation of memory cells.^457,458^ Immune cells can switch between different metabolic states, preferentially using different substrates (glucose, amino acids, and fatty acids) to maintain various functions of specific effectors.^456^ Cellular metabolism controls the function of immune cells by controlling key metabolic nodes, bringing new paradigms to immunology and prospects to treat inflammatory diseases and autoimmune diseases through metabolism. Mills et al. demonstrated that activated macrophages experience substrate alteration for ATP production from OXPHOS to glycolysis, concurrent with elevated ROS and succinate levels that triggers a pro-inflammatory cell state.^459^ Tomas et al. demonstrated that the risk stratification of low- and high-risk plaque in atherosclerosis links the macrophage infiltration to altered mitochondrial substrate oxidation, that is, low-risk plaque exhibits fatty acid oxidation-prone and downregulated glycolysis or anaplerosis by amino acids, while opposing energy preference in high-risk plaques.^460^ In line with this, a similar finding was found by different investigators^461^ and further evidence associates the regulation of immunometabolism of macrophages with other ageing-related diseases, such as type 2 diabetes^462^ and macular degeneration.^463^ Altogether, these studies suggest that the metabolic reprogramming of immune cells might be a promising therapeutic approach to treat ageing-related diseases.

#### Mitochondrial dysfunction

In addition to their roles in cellular energy metabolism and apoptosis, mitochondria are thought to be the central hub of the innate immune response,^464^ that is, mitochondria can not only act as the platform of immune response adaptors (such as MAVS and NLRP3), but also participate in the immune response by producing ROS. During ageing, mtDNA, Tfam, ROS, ATP, cardiolipin, and N-formyl peptide can be released as DAMPs and are recognized by pattern recognition receptors upon mitochondrial stress or compromise, leading to the activation of MiDAS and SASP.^44^ A recent study showed that T cells with dysfunctional mitochondria promoted ageing in mice via inducing circulating cytokines. Blocking TNF-α, a critical cytokine released during the T cell metabolic failure, partially rescued the premature ageing phenotype.^465^ Interestingly, He et al. demonstrated age-associated acetylation of NLRP3 in macrophages might cause inflammatory microenvironment and insulin resistance, while enhancing Sirt2 expression diminished inflammation and related disease phenotype by directly deacetylation of NLRP3,^5^ indicating a possible interplay between epigenetic regulation of inflammageing and mitochondrial metabolic reprogramming. Of note, abnormalities between ER–mitochondrial communication (i.e., ER–mitochondrial dysfunction) could be another crucial player mediating the ageing-related inflammatory phenotype in macrophages.^466^ Another study has demonstrated that de novo NAD biosynthesis in macrophages is the key to the homeostasis of the immune response during ageing,^467^ while α-KG repletion could maintain the α-KG pool in aged mice, lower the bodyweight and fat mass, and improve glucose tolerance in mice fed a high-fat diet.^468^ Collectively, these studies implicate that targeting mitochondrial homeostasis in the immune cells may represent a novel avenue to counteract inflammageing and normalize immunometabolism.

#### Serum phosphate

Studies performed over the past few years make it clear that changes in serum phosphate levels have profound effects in mice and human. Changes in extracellular and intracellular phosphate concentration affect glucose metabolism, insulin sensitivity, and oxidative stress in vivo and in vitro. High concentrations of extracellular phosphate are toxic to cells, which induce cell damage and inflammation when precipitated with calcium (to form calciprotein particles that comprised calcium–phosphate crystals and fetuin-A), thus potentially affect ageing processes.^468,469^ Indeed, impaired urinary phosphate excretion increases serum phosphate level and induces an inflammageing phenotype in mice, and calciprotein particles are found in patients with chronic kidney disease associated with a (mal)adaptation of the FGF-23-Klotho endocrine system.^469^ Direct evidence shows that knocking out fibroblast growth factor-23, a regulator of phosphate homeostasis, causes hyperphosphatemia and vascular calcification in mice, concurrent with multiple ageing-like phenotypes.^470^ Deletion of *Klotho* exhibits similar abnormalities, both could be alleviated by resolving phosphate retention (e.g., on a low-phosphate diet), indicating an underlying link between phosphate and inflammageing. In line with this, augmented circulating levels of calciprotein particles are detected in concert with an increase in serum phosphate and age, which correlate positively with vascular stiffness and chronic sterile inflammation, suggesting that calciprotein particles may be an endogenous pro-ageing factor.^471^ Furthermore, old mice showed muscular degeneration correlated with high serum phosphate concentration and increased levels of integrin-linked kinase and p53, indicative of a possible mechanism in developing sarcopenia.^472^ Another study confirms that hyperphosphatemia induces endothelin-1-mediated senescence in human endothelial cells.^473^ In contrast, Klotho deficiency-induced heart ageing is independent of phosphate metabolism since *Klotho*-mutated mice exhibit a normal range of serum phosphate.^474^

#### One-carbon metabolism

One-carbon (1C) metabolism formed by three reactions, namely the folate cycle, the methionine cycle, and the trans-sulfuration pathway, is of great importance to nucleotide biosynthesis, amino acid homeostasis, methylation modifications, and redox balance.^475^ Recent advances reveal that 1C metabolism is also associated with inflammageing. Perturbations in 1C metabolism have been linked to a biased pro-inflammatory state and may also increase the risk of age-related diseases.^476^ Indeed, a specific deficit in the induction of enzymes of 1C metabolism leads to the accumulation of impaired naive T cells in aged mice, which could be rescued by the addition of products of 1C metabolism (formate and glycine) to the cells.^477^ 1C units in the methionine cycle support the generation of SAM thereby a high SAM:S-adenosylhomocysteine (SAH) ratio, leading to enhanced histone H3 lysine 36 trimethylation and IL-1β expression in macrophages.^478^ Conversely, serine deprivation lowers IL-1β production and inflammasome activation, and alters the transcriptomic and metabolic profile in M1 macrophages via inhibited mTOR signaling.^479^ Congruently, elevated homocysteine, SAM, and SAH levels in plasma are more susceptible to cardiometabolic syndromes.^480^ Moreover, defect of complex I decreases mitochondrial 1C NADPH production, which is associated with increased inflammation and cell death.^481^ These findings highlight a potential regulatory mechanism for 1C metabolism to modulate the inflammatory response. Notably, anti-ageing mechanisms by which metformin can activate the gerosuppressor/tumor suppressor AMPK. One such mechanism may be 1C metabolism that drives the de novo synthesis of purine nucleotides (e.g., AMP), suggesting that de novo biosynthesis of purine nucleotides, which is based on the metabolism of 1C compounds, is a new target for metformin’s actions in ageing.^482^ Therefore, quantification and intervention of 1C metabolism metabolites may open a new avenue to further our understanding of inflammageing.

#### Microbial burden

Ageing is correlated with a reduced abundance of salutary commensal microbes that maintain the intact intestinal barrier and limit the amplification of pathogenic commensals by secretion of specific metabolites, for instance, the short-chain fatty acids.^483^ Increased attention has been paid to microbial homeostasis that connects microbiota to the functionality of multiple organs during ageing. Indeed, gut microbial dysbiosis links to aberrant immune responses and miscommunication between gut microbes and innate immune response may cause various diseases.^484,485^ One study implicates that microbiota-induced IFN-Is instruct a poised basal state of dendritic cells.^486^ Lloyd and Marsland propose that microbes in the host are crucial to establishing lung homeostasis and are affected by ageing to reshape the immune network during chronic inflammation.^487^ Ang et al. found that the ketogenic diet changed the landscape of gut microbiota by reducing intestinal pro-inflammatory Th17 cells, suggesting diet interventions could be a possible means to attenuate ageing and related metabolic diseases.^488^ Similarly, other studies have testified interactions between gut microbiota with cerebral and cardiovascular diseases.^489,490^ In addition, based on deep learning techniques such as neural networks, Galkin et al. established a mathematical model that can reflect the relationship between the microbiological profiles of gut microbiota and the biological age of host, using >3000 microbiome samples from 1165 healthy individuals. They concluded that 39 microbiota taxa were extracted as intestinal markers to best correlate physiological age.^491^ In sum, these studies highlight the importance of microbes in regulating organ function and potential effects on immunosenescence.

### Metabolic regulation of protein homeostasis in ageing

Protein quality control in living organisms is essential for the maintenance of cell viability that maintains a relatively balanced state of the proteome in regards to synthesis, folding and unfolding, modification, trafficking, and degradation of specific proteins.^492^ Protein homeostasis in cells is mainly coordinated by the molecular chaperones, and two proteolytic systems, namely the ubiquitin–proteasome system and autophagy–lysosome system. The imbalance of protein homeostasis is one of the crucial factors causing ageing and the development of ageing-related diseases.^493^ Cell senescence can be accelerated when various organelles meet overloaded stress or proteotoxicity, such as misfolding of nuclear, ribosomal, peroxisomal, and mitochondrial proteins,^494–497^ leading to aberrant protein aggregates that dampen the cross talk of organelles. Accordingly, several cellular hallmarks of proteotoxic stress have been identified, including dysregulated mTOR signaling and autophagy, compartment-specific unfolded protein response in mitochondria (UPR^mt^), and in the endoplasmic reticulum (UPR^ER^).

#### mTOR

The evolutionarily conserved mTOR is a serine/threonine kinase that participates in the central regulation of nutrient sensing, stress response, and longevity.^498^ Two mTOR complexes, namely mTORC1 and mTORC2, have been extensively studied, and both exert their functions by other kinases, such as S6 kinase and Akt.^499^ Park et al. demonstrated that mTORC1 could influence integrated stress response via augmenting activating transcription factor 4 (ATF4) at the posttranscriptional level by 4E-BP1.^500^ Zhang et al. revealed that mTORC1 enhances the protein synthesis capacity, while concordantly inhibiting autophagy, promoting the production of proteosomes via the activation of Nrf1.^501^ Conversely, other studies suggested mTORC1 inhibition induced an increase in proteasome abundance and protein degradation.^502,503^ mTORC2 has been demonstrated to inhibit chaperone-mediated autophagy during ageing that can be restored by phosphatase PHLPP1, thereby representing a lysosomal pathway to counteract age-associated dysfunction of protein degradation.^504^ Moreover, rapamycin-induced insulin resistance is triggered by the inhibition of mTORC2, although it is irrelevant with longevity.^505^

#### Autophagy

Impairment in autophagy has linked to the derailment of proteostasis that is closely associated with ageing and age-related diseases, including macroautophagy, microautophagy, and chaperone-mediated autophagy. A significant effort has been made to elaborate on the role of autophagy in the field of cerebral proteinopathies, including Huntington’s disease (HD), AD, PD, and amyotrophic lateral sclerosis (ALS).^506–508^ Sorrentino et al. reported that enhancing mitophagy helped resolve amyloid-β proteotoxicity in mouse and *C. elegans*, providing a promising strategy targeting mitochondrial proteostasis to delay AD.^509^ Poewe et al. showed that α-synuclein aggregation and clearance of undegraded proteins were therapeutic targets for PD.^510^ Similarly, enhancement of cytosolic proteostasis pathways (through autophagy or proteasome) shows promise for HD and ALS treatments.^511,512^

Moreover, identifying novel mitophagy receptors or booster of mitophagy further enhances our understanding of the complexity of mitochondrial quality control. Li et al. showed that mitophagy receptor protein FUNDC1 located at the mitochondrial outer membrane that is associated with cytoplasmic chaperone protein HSC70, leading to the recruitment of damaged or misfolded cytosolic proteins to mitochondria, and then shuttle to the mitochondrial matrix through the TOM–TIM complex, culminating with the degradation by matrix-localized mitochondrial protease LONP1.^513^ Ryu et al. identified the natural compound urolithin A as a mitophagy inducer, which enhanced mitochondrial and muscle function.^514^ We and others have found the potential therapeutic benefits by PGC1α and TFEB upregulation in counteracting declined autophagy capacity and reduced proteostasis.^362,515^ Together, these findings indicate a regulatory effect of autophagy in protein homeostasis necessary for proper organ function, especially in the brain and other high energy-demanding organs.

#### UPR^mt^

UPR^mt^ is one key mechanism in mitochondrial quality control involving multiple components, such as chaperones, proteases, and other stress sensors and effectors.^516^ Apart from cell respiration and ATP production, mitochondria also participate in immune response, cell senescence, and apoptosis. Notably, most proteins required by the processes above are translated from nuclear-encoded genes; thus, it is crucial to orchestrate mitochondrial–nuclear communication that ensures a precise transcriptional response by the UPR^mt^. Indeed, a plethora of defects within aged mitochondria that activate the UPR^mt^ implicates intrinsic surveillance of mitochondrial function being reliant on mitochondrial stress response.^517^ Zhao et al. discovered that the accumulation of unfolded proteins in the mitochondrial matrix resulted in transcriptional upregulation of nuclear-encoded mitochondrial stress genes, including hsp60, hsp10, mtDnaJ, and ClpP, without interfering gene expression that was related to endoplasmic reticulum (ER) stress. Teske et al. demonstrated that CHOP-induced ATF5 to trigger cell death in response to perturbations in protein homeostasis.^518^ Notably, a CHOP element, coupled with the C/EBP domain, was found in the gene promoter regions.^519^ Further analysis revealed that c-JNK could bind to an AP1-binding site within the promoter regions of CHOP and C/EBP, underscoring an indispensable role of the JNK signaling pathway in UPR^mt^.^520^ Furthermore, using the nematode model, transcriptional factors DVE-1 and ATFS-1, along with H3K27 demethylases jmjd-1.2 and jmjd-3.1 and other factors, have been identified as UPR^mt^ activators.^521–523^ Although ample studies provide a framework for the signal transduction of UPR^mt^, the exact and complete molecular mechanism that cells (especially in mammals) sense mitochondrial stress signals and transmit the signals to the nucleus await further elucidation.

#### UPR^ER^

Response to ER stress caused by unfolded or misfolded proteins (UPR^ER^) represents another intrinsic mechanism to counteract multiple stress stimuli that leads to perturbed proteostasis. The UPR^ER^ contains three upstream stress sensors in the ER membrane, i.e., inositol-requiring enzyme-1, PKR-like ER kinase, and ATF6, which is activated by the presence of unfolded proteins within the ER, ensuring signal transduction to the well-known downstream effector molecules.^524^ UPR^ER^ compromise lies at the nexus of inflammageing and metabolic dysfunction, while its constitutive activation attenuates multiple age-related diseases and extends longevity.^525^ Most of the pioneering findings regarding UPR^ER^ are derived from the studies using worms. Taylor and Dillin revealed how to rescue declined UPR^ER^ in the ageing of *C. elegans* and found that age-onset loss of ER proteostasis could be restored by neural expression of a constitutively active form of X-box-binding protein 1.^526^ Similarly, Daniele et al. facilitated UPR^ER^ specifically in neurons to promote ER function by lipid depletion through lipophagy. Interestingly, this beneficial metabolic alteration is independent of chaperone induction, but is regulated by EH domain-binding protein 1-mediated lipophagy, thereby enhancing organismal stress resistance and extending the life span.^527^ Schinzel et al. utilized a whole-genome CRISPR-based knockout screen to identify transmembrane protein 2 (TMEM2), a hyaluronidase that degraded hyaluronan in the extracellular matrix, was capable of modulating ER stress through CD44, ERK, and p38. Ectopic expression of human TMEM2 promoted ER homeostasis and extended longevity in *C. elegans*, indicating a conserved health-promoting pathway via elevating the TMEM2–UPR^ER^ axis.^528^ Although more efforts are required to illustrate the orthologs of human UPR^ER^ targets and pathways, these findings advance our understanding of how the UPR^ER^ changes with age and how this impacts disease development, thus opening new therapeutic avenues to treat age-associated diseases.

### Metabolic regulation of organelle homeostasis in ageing

Imbalance of cellular metabolism and organelle homeostasis during ageing is one of the major drivers causing ageing-related diseases.^16^ Several aspects that aim to delineate the changes in organelle ageing, in particular, to clarify the causal relationship between organelle dysfunction and ageing and/or degenerative diseases, and to explore new strategies targeting organelle homeostasis to intervene ageing and related disorders at varying levels of metabolism, have been investigated for long and some critical findings are of great importance.^529,530^

#### Mitochondria

For decades, mitochondrial biology lies at the center of mechanistic exploration in metabolic regulation of cell senescence and organismal ageing.^531^ Mitochondrial dysfunction has been linked with mtDNA mutations, aberrant mitochondrial respiration, dynamics, biogenesis, autophagy, and other quality control machinery.^532^ Apart from their roles above in redox balance and immune response, mitochondria also involve in proteostasis and other cellular functions that intensively interact with ER and nucleus.

Guo et al. developed grazing incidence structured illumination microscopy and found most of the mitochondrial fission events, while ~60% of the mitochondrial fusion events actively occurred at the contact sites between mitochondria and ER.^533^ Consistently, Gӧbel et al. demonstrated that mitofusin 2-mediated mitochondria–ER contacts in astrocytes promote repairing the injured brain.^534^ Moreover, reduced mitochondrial fusion and Huntingtin levels were observed in impaired dendritic maturation, thereby leading to behavioral deficits,^535^ indicative of a fundamental role of mitodynamics in tissue homeostasis. Another critical player, mitochondrial permeability, has been manifested as an indispensable regulator of mitochondrial function. VDAC oligomerization warranted an increased mitochondrial outer membrane permeability, which was essential to trigger released mtDNA-induced inflammatory response.^536^ Ying et al. demonstrated that short-term mitochondrial permeability transition pore opening (aka mitoflash) induced a nuclear reprogramming by histone methylation, suggesting a novel mitochondrial regulation of nuclear epigenetics.^537^ Besides, MacVicar et al. identified that the mTORC1–LIPIN1–YME1L regulatory axis was a posttranslational regulator of mitochondrial proteostasis at the interface between metabolism and mitochondrial dynamics.^538^

#### Lysosome

Lysosomes are crucial cellular organelles for human health that function in digesting and recycling of extracellular and intracellular macromolecules. Given that many neurodegenerative diseases (e.g., PD and AD), as well as reduced life span, are linked to lysosomal dysfunction, enhancing lysosomal function becomes an option to promote health span in certain circumstances, such as reduced ability to clear protein aggregates during neural stem cells ageing. Leeman et al. demonstrated that aged neural stem cells exhibited decreased lysosomal genes, thereby a slower clearance of protein aggregates. Normalization of lysosomal function in these cells could restore the defects and enhance the quiescent neural stem cell activation.^539^ Castellano et al. investigated the metabolic cue that activated mTORC1 at lysosome and found that SLC38A9-mediated import and Niemann–Pick C1-mediated export of lysosomal cholesterol were the drivers for mTORC1 activation.^540^ Further analysis found that arginine was essential for mTORC1 activation and represented as the lysosomal messenger sensed by SLC38A9.^541^ Via developing a method for rapid lysosomal isolation and metabolomics, Abu-Remaileh et al. manifested that mTOR inhibition decreased the lysosomal efflux of most essential amino acids in a V-ATPase-dependent manner.^542^ Moreover, overexpression of the lysosomal biogenesis regulator TFEB homolog (HLH-30) in worms has been proven beneficial. It may prolong life span through repression of mTOR signaling and engagement of autophagic processing.^543^ Collectively, these results reinforce the concept that regulation of lysosomal function is one of the most efficient anti-ageing interventions.

#### Peroxisome

Peroxisomes are dynamically and metabolically active that signal various intracellular events, therefore exhibiting a high degree of plasticity, linking redox balance, and nutrient digestion to immune metabolism. They are capable of autonomous replication via fission. Recent studies have revealed that beyond the essential roles in fatty acid oxidation, anaplerotic metabolism, and production and scavenging of ROS, peroxisomes also participate in the metabolic regulation of cell senescence.^544^ Evidence shows the significant role of weakened peroxisomal functions, including diminished peroxisomal autophagy (pexophagy), loss of communication with ER and mitochondria, and dysregulation of peroxisomal proteostasis in accelerated ageing and related diseases.^496^ Peroxisomes not only eliminate (mainly detoxify by peroxiredoxins, glutathione peroxidases, and catalases), but also produce (mainly oxidize by xanthine oxidases and acyl-CoA oxidases) H_2_O_2_. Consequently, peroxisomes may serve as a direct source of oxidative stress, especially during ageing, by decreased peroxisome turnover and downregulated detoxifying enzymes.^545^ Dixit et al. revealed that the well-known mitochondrial antiviral signaling protein, MAVS, was also a peroxisome-localized adaptor that functioned in innate antiviral immunity.^546^ The tuberous sclerosis complex has also been found on the peroxisome to inhibit mTORC1 in response to endogenous ROS.^547^ Similar findings were reported that a portion of extranuclear ATM was localized to peroxisomes and mediated ROS-induced pexophagy,^548^ while AMPK and dietary restriction extended the life span of *C. elegans* via coordinating mitochondrial and peroxisomal homeostasis.^3^ Of note, the peroxisomes remain the least understood of all organelles, yet accumulating studies uncover their roles in regulating metabolism and ageing in concert with mitochondria, ER, and other organelles. Further investigations on peroxisomal quality control warrant a deeper understanding of their roles in cellular homeostasis.

Together, comprehensive mapping of metabolic networks should help dissect the interaction and cross talk among different organelles, furthering our understanding of the molecular and cellular basis of organelle ageing.

### Novel interventions and techniques in metabolic ageing research

Increasing attention has been focused on depicting the metabolic signatures of cells from single-cell resolution, as well as the whole metabolic landscape of organisms in a dynamic manner. In particular, identifications of age-related metabolic phenotypes or biomarkers that are closely correlated with overall fitness are increasingly being recognized as therapeutic targets. The discovery of senescence-related genes and metabolites by combining transcriptomics, proteomics, epimicromics, secretomics, metabolomics, phenomics, and large population cohort studies sheds light on the complex senescence signaling networks. Among the multi-omics, single-cell RNA sequencing is the hotspot to analyze the molecular mechanism of organ ageing.^549^ For example, Wang et al. compared young and aged nonhuman primate ovaries using single-cell transcriptomics. In-depth analyses of gene expression dynamics of oocytes revealed oxidative damage was an important determinant in ovarian ageing.^550^ Bian et al. characterized a comprehensive map of human macrophage development at single-cell resolution, providing a deeper understanding of the functions of tissue-resident macrophages.^551^ In addition, the advances in metabolomics, especially single-cell metabolomics and their applications, further add strength to the field of clinical pharmacology.^552,553^

Continuous focus on the clearance of intracellular senescence-inducing factors is a relevant direction for treating age-associated disorders. Many reports manifest that removal of senescent cells (e.g., via senolytics) or abnormally active transposons (e.g., via inhibitors to LINE1 transposons) from ageing animal models is salutary.^7,40,112,554^ Conversely, the repletion of rejuvenating factors (NAD^+^) also improves various organ functions, which have been experimentally proven through genetic manipulation or diet/supplement intervention, although detecting NAD^+^ levels in vivo remains challenging. Genetically encoded fluorescent probes represent promising tools to realize high-dynamic, high-resolution, high-throughput NADH/NAD^+^ detection in vivo, empowering a real-time visualization of compartmentalized NAD^+^ that facilitate screening for NAD-associated drug design or gene candidates identification.^363,374^ Furthermore, manipulation of melatonin,^555^ supplementation of compounds, such as spermidine,^556^ acarbose,^557^ and urolithin A^514^ may also exert an anti-ageing effect through metabolic remodeling. Intriguingly, a controversial study using parabiosis demonstrated that the enhanced level or administration of GDF11 could correct DNA damage accumulated in ageing mouse satellite cells,^558^ while its paralog myostatin regulates energy homeostasis in the heart and prevents heart failure.^559^ Although heterochronic parabiosis of young and old mice implies the potential to attenuate the ageing process and improve cell function, how to rejuvenate senescent cells via autologous rather than allogeneic resources remains a central question to avoid issues regarding immunity and ethics.

**Fig. 7:**
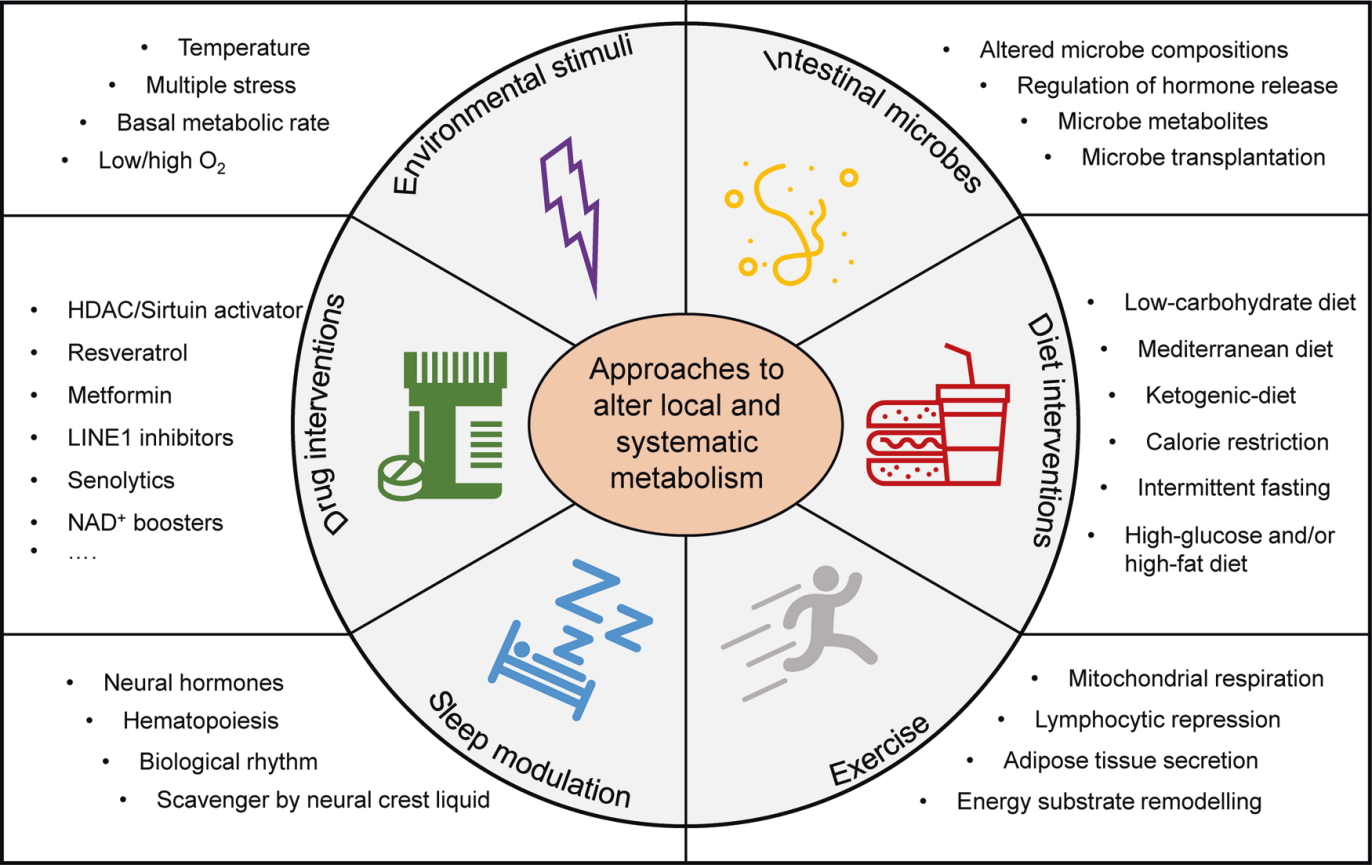
Approaches to alter local and systemic metabolism. Six aspects, including environmental stimuli, sleep modulation, exercise, intestinal microbes, diet, and drug interventions, may reprogram the metabolism thereby benefiting to healthy ageing

CR, drug intervention, microbial regulation, and sleep/exercise represent simple but also noninvasive approaches to extend health span and ameliorate age-related pathologies, at least, via enhancing the tissue function across a broad spectrum of animals (Fig. 7). The level of CR is indeterminate from individual-by-individual, and long-term CR sounds impracticable to the general public. In contrast, resveratrol, a naturally occurring small molecule that induces metabolic benefits resembling CR, increases mitochondrial biogenesis, partly through activation of SIRT1 and PGC1α.^560^ Similarly, metformin, a hypoglycemic agent widely applied in type II diabetes mellitus, has been found to extend longevity and health span in animal models.^178,561^ Rapamycin also exerts a similar protective response in *C. elegans* and mice.^562^ Such an endeavor is worth trying that directs strategies to mimic the life-extending effect using known compounds and pathways. In addition, emerging evidence suggests that sleep modulation links haematopoiesis to atherosclerosis,^563^ while the development of microbiota-based interventions, such as probiotics and prebiotics extended health span.^564^ Physical exercise also gives considerable benefits in improving energy metabolism, yet this is unsuitable to the population with severe conditions, such as cardiovascular diseases, asthma, osteoarthrosis, diabetes, etc., which predominantly happen among the aged.

## Conclusive remarks

**Fig. 8:**
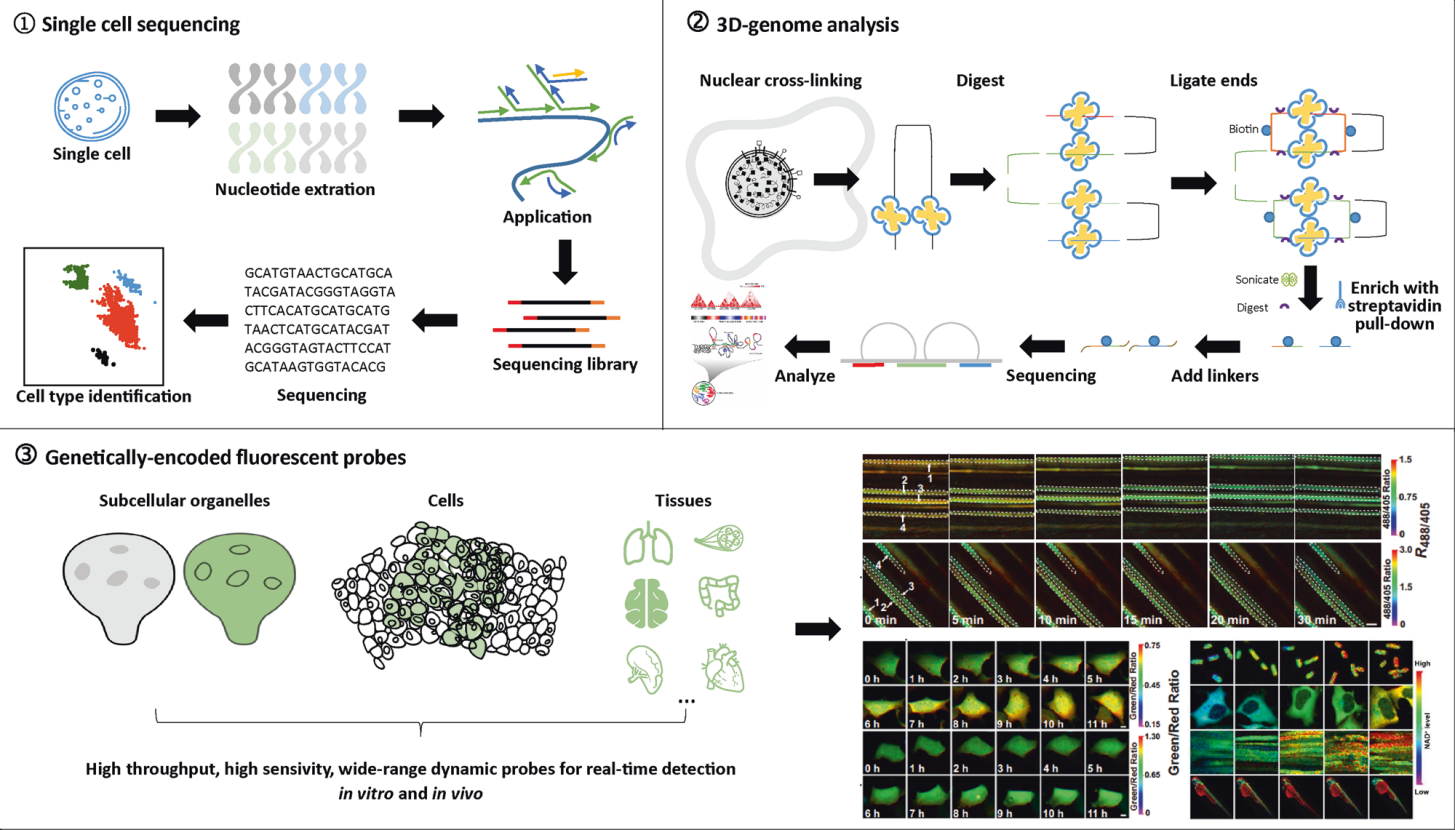
Novel techniques accelerate the discovery of anti-ageing mechanisms and therapies. Illustrations of three advanced approaches, i.e., single-cell sequencing, 3D-genome analysis, and genetically encoded fluorescent probes in dissecting genetic, epigenetic, and metabolic alterations in ageing and anti-ageing research

Many conventional drugs have been found to exert an anti-ageing effect. Thus, developing methods aiding high-throughput screening of anti-ageing drugs from currently approved drug libraries is cost-efficient to promote pharmacological interventions of ageing, which required state-of-the-art techniques to delineate the dynamics and interactions of key molecules/metabolites involving the ageing process (Fig. 8). Further elucidation of regulatory mechanisms of organ homeostasis in senescent cells will be informative to anti-ageing agent design.

Apart from practices in assisting medical image diagnosis, pathological classification, and treatment decision-making, machine learning, such as convolution neural network, has been integrated into ageing biology to calculate or predict cell ageing.^565–567^ The algorithm models are continually optimized to define the best cell senescence markers. It is thought the extent of cell senescence or organismal ageing could be readily predicted by certain factors (e.g., inflammatory cytokines or metabolites) in a blood test soon. Such artificial intelligence can also be utilized to pick candidate anti-ageing compounds or drug targets in high-throughput screening, which could lead to disruptive innovation of the pharmaceutical industry and finalizing our goal to health ageing.

In sum, the inflammatory, epigenetic, and metabolic regulation of cellular components and activities is the core event of environmental and genetic manipulations that link cell senescence to organismal ageing. Owing to the significant progress in understanding the aspects above, incremental approaches and concepts that help healthy ageing have been identified, and it will continue to be a prosperous field of research for the next decades to come.
